# Metabarcoding of harmful algal bloom species in sediments from four coastal areas of the southeast China

**DOI:** 10.3389/fmicb.2022.999886

**Published:** 2022-08-31

**Authors:** Zhaohui Wang, Liang Peng, Changliang Xie, Wenting Wang, Yuning Zhang, Lijuan Xiao, Yali Tang, Yufeng Yang

**Affiliations:** ^1^College of Life Science and Technology, Jinan University, Guangzhou, China; ^2^Southern Marine Science and Engineering Guangdong Laboratory, Zhuhai, China

**Keywords:** eukaryotic algae, 18S rDNA, human activities, resting stages, the Yellow Sea, the East China Sea

## Abstract

In the past three decades, harmful algal blooms (HAB) have become more frequent and widespread in southeast Chinese sea areas. Resting stages are regarded as the “seed bank” of algal blooms, and play an important role in initiating HABs. The distribution of resting stages in sediments especially those of HAB species can make good predictions about the potential risk of future blooms, however with limited reports. In this study, surface sediment samples were collected in the four sea areas along the southeast Chinese coasts, including Dafeng Port (DF) in the southern Yellow Sea, Xiangshan Bay (XS), Funing Bay (FN), and Dongshan Bay (DS) in the East China Sea. Diversity and community structure of eukaryotic microalgae in surface sediments were assessed by metabarcoding V4 region of the 18S rDNA, focusing on the distribution of HAB species. Biogenic elements including total organic carbon (TOC), total nitrogen (TN), total phosphorus (TP), biogenic silicon (BSi), and moisture content (MC) were analyzed. A total of 454 eukaryotic algal OTUs were detected, which belonged to 31 classes of 9 phyla. Altogether 149 algal species were detected in this study, and 59 taxa have been reported to form resting stages. Eukaryotic algal community was similar in XS, FN and DS of the East China Sea, which were predominated by dinoflagellates. However, algal community was different in DF of the Yellow Sea, and characterized by the dominance of chrysophytes and low OTU richness. The distribution of most abundant HAB species showed positive correlations with TN, BSi, and TOC, suggesting that eutrophication and consequent increase in diatom productivity may have a significant influence on the distribution of HAB species and facilitate the occurrence of HABs. Furthermore, HAB species occurred more abundantly and widely in FN. Our results suggest high potential risks of HABs in the southeast Chinese coast especially in Funing Bay.

## Introduction

Harmful algal blooms (HABs) are caused by massive proliferation of certain algae in the marine environment, which pose threats to ecological security, directly or indirectly to human health, and to local social and economic development (Granéli et al., 2008). In recent decades, HABs have evolved into frequent abnormal ecological disasters under the influence of intensive human activities and climate changes (Anderson et al., 2002; Hallegraeff et al., 2021).

Many planktonic microalgal species have been reported to produce dormant stages as a part of their life cycles (Head, 1996; Von Dassow and Montresor, 2011) in the forms of resting spores and resting cysts (hereafter referred to as resting stages). Once the resting stages formed, they sink to the sea floor and become a part of the benthic assemblages until hatching occurs (Head, 1996). The germination of resting stages in sediments can provide a large number of vegetative cells to the water column within a short time and promote the occurrence of blooms (McQuoid, 2002). Although only a small part of phytoplankton can form resting stages, many toxic and HAB species have been reported to form resting stages, especially those causing recurrent blooms (Bravo and Figueroa, 2014). Resting stages are regarded as the “seed bank” of algal blooms, and play an important role in initiating HABs (Smayda, 2002; Anderson et al., 2014; Kim et al., 2020; Castaneda-Quezada et al., 2021). Studies of phytoplankton resting stages are useful to trace present and predict future blooms or toxic events of a certain species in a particular area (Anderson et al., 2014; Kim et al., 2020).

The identification and quantification of the resting stages of phytoplankton have been traditionally based on microscopic observations and cell counting after separating cells from sediments. Microscopic observations require professional taxonomic expertise for distinguishing the tiny morphological differences among species (Shang et al., 2019). Furthermore, only a small proportion of resting stages are known for their corresponding vegetative cells and vice versa. Misidentification is thus common and almost unavoidable due to these difficulties (Gómez et al., 2017; Shang et al., 2019). Advances in the sequencing of DNA extracted from natural water and sediment samples offer huge opportunities for biodiversity monitoring and assessment (reviewed by Lear et al., 2018). DNA sequence analysis using metabarcoding has been widely applied in characterizing the biodiversity and community structure of microeukaryotes in marine sediments (Gran-Stadniczeñko et al., 2019). This method provides information on many organisms affiliated with several trophic levels in a biological system (Lanzén et al., 2016), some of which are difficult to identify with classic methods, hard to culture, fragile, and rarely analyzed (Egge et al., 2015). Metabarcoding offers the prospect for determining the distribution and diversity of microeukaryotes in the world’s oceans and enables a more complete assessment of anthropogenic effects on ecosystems (Lekang et al., 2020), particularly for unicellular microalgae that often lack distinctive morphological characteristics (Vaulot et al., 1989) and have complex life cycles (Von Dassow and Montresor, 2011).

The Chinese southeast coast is one of the most rapid development regions in China the past three decades. With the economic development and population increase, the coastal waters are increasingly affected by human activities. Meanwhile, the coastal China seas have experienced a long-term surface warming since the late 1950s particularly along the East China Sea (Cai et al., 2016). HABs have become more frequent and widespread in southeast Chinese sea areas driven by climate change and human impacts (Chen et al., 2010; Yu et al., 2020; Gu et al., 2022). At present, most studies on HABs in southeast Chinese coastal areas are focused on the Changjiang Estuary and its surrounding waters, which is the most notable coastal region for frequent HAB occurrences in China since 2000 (Yu et al., 2017; Gu et al., 2022). However, phytoplankton community and HAB studies in other sea areas along the Chinese southeast coasts were limited reported.

In this study, metabarcoding based on high-throughput sequencing (HTS) was used to analyze the biodiversity and distribution of eukaryotic microalgae in surface sediments from four sea areas along the southeast Chinese coasts, i.e., Dafeng Port in the southern Yellow Sea, and Xiangshan Bay, Funing Bay, and Dongshan Bay in the East China Sea. Our study aimed to assess the diversity and distribution of benthic microalgae by metabarcoding with a focus on the phytoplankton resting stages; to understand the distribution of HAB species in surface sediment; and to discuss the potential risk for HABs in the four sea areas. As far as we know, it is the first report of metabarcoding study on microalgae community in surface sediments from the four sea areas.

## Materials and methods

### Studying areas

**FIGURE 1:**
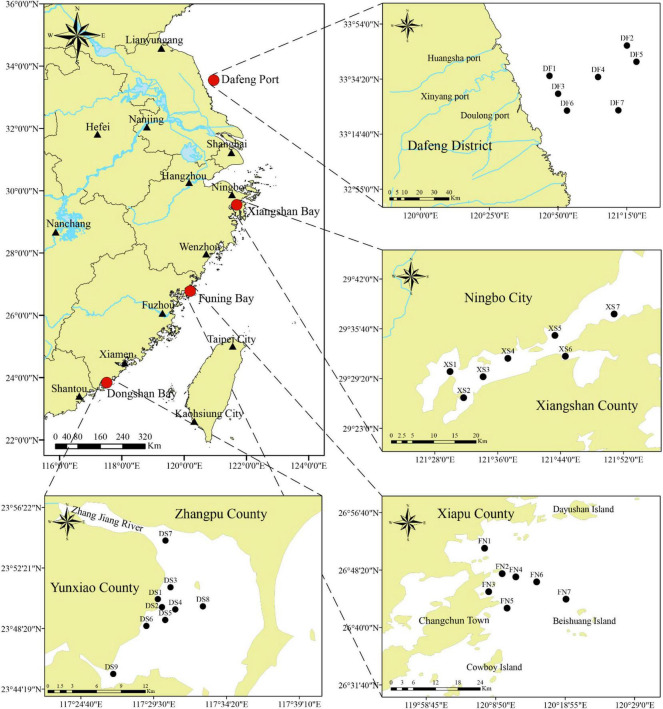
The location of the four sea areas showing sampling stations in this survey. Stations DF1–DF7 are in Dafeng Port, stations XS1–XS7 in Xingshan Bay, stations FN1–FN7 in Funing Bay, and stations DS1–DS9 in Dongshan Bay.

Surface sediments were collected from four sea areas along the southeast Chinese coasts, in which Dafeng Port (DF, stations DF1–DF7) is located in the southern part of the Yellow Sea, and Xiangshan Bay (XS, stations XS1–XS7), Funing Bay (FN, stations FN1–FN7) and Dongshan Bay (DS, stations DS1–DS9) are located in the East China Sea (Figure 1).

Dafeng Port is an important port in Jiangsu Province, China. The first phase of Dafeng Port started construction at the end of 1998, and two 10,000-ton berths were completed and put into operation in October 2005. Dafeng Port has operated international container liner lines to Incheon Port and Busan Port of Korea, and the Europe and America lines *via* Shanghai Port since September 2007. The second and third phases of Dafeng Port project were completed and put into operation in November 2009 and March 2014, respectively. Dafeng Port has an annual handling capacity of 50 million tons and 1 million twenty-foot equivalent unit (TEU) containers in 2015 (Xu, 2021). It is also a famous offshore wind farm in Central China. Dafeng Port undertakes numerous economic and ecological functions, including shipping, mariculture, fishery infrastructure, wind farm and wetland reservation. Water quality in Dafeng Port has been deteriorating in recent two decades, and phytoplankton biomass has increased (Zhang et al., 2015).

Xiangshan Bay locates in the southeast of Ningbo city, Zhejiang Province, China. It is a long and narrow semi-closed bay from southwest to northeast (Figure 1). It is the largest aquaculture base in Zhejiang Province, including fish cage farms, floating raft kelp cultivation, and Seine breeding for shrimp and crab (Xu et al., 2021). Two thermal power plants locate at the bottom and central bay, respectively, which influences phytoplankton community through cooling water discharge due to the low water exchange (Li et al., 2014; Luo et al., 2018). The eutrophication problem in Xiangshan Bay has become increasingly serious with the development of industry, agriculture and the impacts of human activities during the recent decades (Dong, 2012; Wu et al., 2017). Meanwhile, algal blooms occurred more frequently, and 16 bloom events were recorded between 2010 and 2018 including those of *Chaetoceros curvisetus, Skeletonema costatum*, *Prorocentrum donghaiense* [currently as a synonym of *Prorocentrum shikokuense* (Takano and Matsuoka, 2011)], *Karenia mikimotoi*, *Noctiluca scintillans*, etc. (Feng, 2010; Dong, 2012; Qi, 2021; Xie et al., 2021).

Funing Bay is located in the northeast of Fujian Province, China. It has a subtropical monsoon climate with clear seasonal changes. It is one of the most important fishing areas with rich fishery resources and high productivity in southern Chinese coast (Xie, 2018). Funing Bay is also an important mariculture area, and aquaculture products include fish, shrimp, crab, and shellfish. The phytoplankton community in Funing Bay is characterized by rich species richness and high density, dominated by diatoms such as *S. costatum*, *Pseudonitzschia* spp. etc. However, dinoflagellate blooms occurred frequently as well including blooms of *P. shikokuense*, *N. scintillans*, *Alexandrium tamarense*, *Scrippsiella acuminata* (formerly *Scrippsiella trochoidea*), and *K. mikimotoi* (Chen et al., 2004; Liu et al., 2008; Quan et al., 2015; Liang et al., 2019). Paralytic shellfish poisoning (PSP) toxins in shellfish were contaminated to a certain degree (Liang et al., 2019), and human diseases and even deaths caused by eating contaminated shellfish occurred from time to time (Guo, 2012).

Dongshan Bay is located in the southeast of Fujian province, China. It is a semi-enclosed bay in the transition area between the East China Sea and the South China Sea (Wu, 2021). Dongshan Bay is an important fish cage area in Fujian Province, as well as macroalgae and shellfish cultivation (Sun L. et al., 2017). Algal blooms recorded in Dongshan Bay are mostly those of diatoms, however toxic and harmful dinoflagellates have been frequently recorded in water samples, such as *K. mikimotoi*, *Lingulodinium polyedrum*, and *Dinophysis caudata* (Wang, 2020). PSP was detected frequently in shellfish samples collected from Dongshan Bay (Wang, 2020), and human poisoning events occurred after eating PSP contaminated shellfish (Chen et al., 2018).

### Collection of sediment samples

Surface sediments were collected from 7–9 stations in each sea area (Figure 1) using a Peterson grab between 2014 and 2018, i.e., November 2016 in Dongshan Bay, April 2018 in Funing Bay, July 2016 in Xiangshan Bay, and November 2014 in Dafeng Port. The top 2 cm of sediments were sampled with a polyethylene spatula, and placed in a sealed glass vial, and then stored in −80°C for further treatments. Before DNA extraction, the sediment samples were stored under dark conditions at low temperatures (4–8°C) for 6 months to inactivate most of the microalgal vegetative cells and to ensure that the algal DNA extracted from the sediment samples mostly belonged to the resting stages (Piredda et al., 2017).

### DNA extraction, polymerase chain reaction amplification, and sequencing

About 0.5 g of sediment sample was applied for environmental DNA (eDNA) extraction using Qiagen’s PowerSoil DNA Isolation kit (Qiagen, Germany) according to the manufacturer’s instructions. The extracted DNA was quantified and checked using a Nanodrop ND-1000 Spectrophotometer (NanoDrop Technologies, Wilmington, DE, United States), and a highly pure genomic DNA (260/280 nm≈1.8) was used for Illumina sequencing.

The V4 region of the 18S rDNA was amplified using the universal eukaryotic primers 3NDf 5′-GGCAAGTCT GGTGCCAG-3′ (Cavalier-Smith et al., 2009) and V4_euk_R2 5′-ACGGTATCTATCTCTTCG-3′ (Bråte et al., 2010). The polymerase chain reaction (PCR) mixtures (20 μl final volume) contained 10 ng of total DNA template, 0.8 μl each primer (5 μM), 2 μl 2.5 mM dNTPs, 4 μl 5× of FastPfu Buffer, 0.4 μl FastPfu Polymerase, and 0.2 μl BSA, adding ddH_2_O to 20 μl. PCR reactions were as follows: initial denaturation at 95°C for 3 min, followed by 35 cycles of 30-s denaturation at 95°C, annealing at 55°C for 30 s, extension at 72°C for 45 s, and a final 10-min extension at 72°C. PCR products were run on a 2% agarose gel to check amplicon lengths. For each sample, multiple PCR replicates were pooled and purified using the GeneJET Gel Extraction Kit (Thermo Scientific). The PCR products (ca. 450-bp) were then sequenced on an Illumina MisSeq PE300 platform (Illumina, San Diego, CA, United States). Sequencing was performed by Majorbio Bio-pharm Technology Co., Ltd. (Shanghai, China).

### Bio-information analyses

Raw data from the Illumina libraries were assigned to individual samples by their barcodes. Paired-end reads were truncated to remove unique barcode and primer sequences, then assembled using FLASH (Version 1.2.7). The obtained filtered sequences with error were removed *via* using QIIME (version 1.8^1^). Then an Operational Taxonomic Unit (OTU) table was generated at 97% sequence similarity by using UPARSE (version 7.1^2^) after removal of singletons and doubletons. The taxonomic assignment of each OTU was classified against the SILVA 18S rRNA database (release138^3^), and further annotated against the NCBI database^4^ (searched on 17 April 2022). The raw data were deposited into the National Center for Biotechnology Information (NCBI) Sequence Read Archive (SRA^5^) with the accession number PRJNA837864. The classification of algal OTUs was confirmed by AlgaeBase (Guiry and Guiry, 2022).

### Analyses of biogenic elements

Sediments for biogenic elements analysis were dried in an oven at 40°C until a constant weight was reached, and the moisture content (MC) of the sediments was calculated. The dried sediments were ground gently with an agate mortar and pestle, sieved through a 100 μm-mesh for homogenization. Total organic carbon (TOC) and total nitrogen (TN) was measured by a Perkin-Elmer 2400 Series II CHNS/O Analyzer (Perkin Elmer Inc., United States). Total phosphorus (TP) was measured by potassium persulfate digestion method (Thien and Myers, 1992). Biogenic silica (BSi) was measured by the molybdate blue spectrophotometric method after removing the carbonates and organics by 1 mol/L HCl and 10% H_2_O_2_ and digested using 0.5 mol/L Na_2_CO_3_ solution (Mortlock and Froelich, 1989). The quality assurance/quality control (QA/QC) was assessed by the analyses of blank reagents and five replicates of the certified reference material (Offshore Marine Sediment, GBW 07314). The analytical precision was controlled to within 5% for biogenic elements.

### Statistical analyses

Rarefaction curve was generated to assess the degree of sample saturation using picante and vegan function packages of R4.1.0. In order to get the equal value of DNA reads in each sample, DNA reads were normalized to the number of DNA reads in the sample with the fewest reads in this study (28,105 reads), and the normalized data were used for further analyses. The alpha diversity indexes of eukaryotic organisms and eukaryotic algae, including OTU richness, Chao1, Shannon diversity, and Pielou index were calculated. In accordance with their relative abundance, the OTUs were divided into three groups: abundant, intermediate, and rare, accounting for ≥0.1%, <0.1% but ≥0.01%, and <0.01% of the total eukaryotic reads at least one sample, respectively (Mangot et al., 2013; Logares et al., 2014). The Bray-Curtis (BC) index was used as a measure of similarity between the samples (beta-diversity). A distance matrix was computed with the BC index, and a hierarchical cluster tree was constructed using the unweighted pair group method with arithmetic mean (UPGMA) with bootstrap support. Non-metric multidimensional scaling (NMDS) was constructed based on BC index using vegan and ggplot2 packages. Venn diagrams were calculated and plotted based on different samples and sea area using the R4.1.0 package VennDiagram. Phylogenetic trees were generated in MEGA7.0, using the Maximum Likelihood (ML) method with 1000 bootstrap replicates.

Correlations between HAB species and environmental factors were explored using Spearman’s correlation coefficient by R package psych, and the figures were drawn by R package corrplot. The canonical correspondence analysis (CCA) with a Monte-Carlo permutation test was performed using Canoco 4.5 (Microcomputer Power, United States) to reveal the correlations between the eukaryotic algal community and environmental factors following the gradient length of the first axis (value = 4.11 in this study) by detrended correspondence analysis (DCA). All data were logarithmically transformed to obtain the equal weight of the elements in the CCA analysis. The bubble chart was drawn with the R package ggplot2. The histogram and pie figures were drawn with Microsoft Office Excel 2019.

## Results

### Sequence data

**FIGURE 2:**
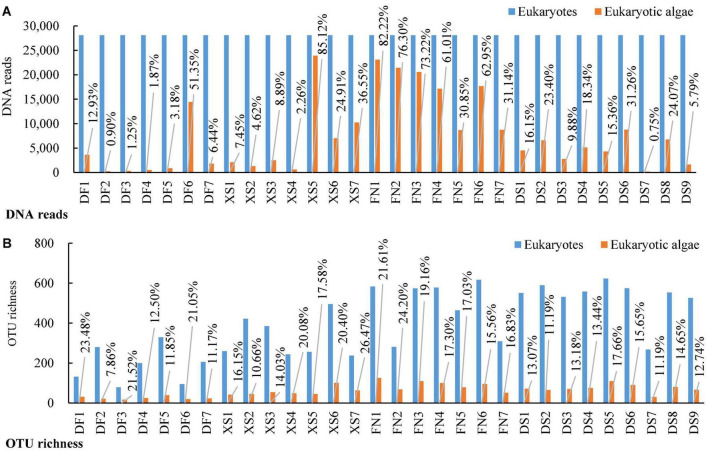
DNA reads and OTU richness of eukaryotic organisms and eukaryotic algae in surface sediments from 30 stations of the four sea areas. **(A)** DNA reads; **(B)** OTU richness. Stations DF1–DF7 are in Dafeng Port, stations XS1–XS7 in Xingshan Bay, stations FN1–FN7 in Funing Bay, and stations DS1–DS9 in Dongshan Bay.

A total of 1,508,569 eukaryotic DNA sequences and 3,899 OTUs were obtained in 30 surface sediment samples from the four sea areas in the southeast Chinese coasts. Rarefaction curve suggested adequate coverage for all datasets (Supplementary Figure 1). DNA reads in each sample ranged from 28,105 at station DS6 to 73,653 at station DF4. After normalized the sequence data with the fewest reads (28,105 reads in this study), 3,738 eukaryotic OTUs were obtained and OTU richness were between 175 and 623 OTUs in each sample, with the lowest at station DF3 and the highest at station DS5, respectively. There were 227,772 algal sequences and 454 algal OTUs. The number of algal sequences at each station varied greatly (Figure 2A), ranging from 211 to 23,922 reads with the highest at station XS5 and the lowest at station DS7. The proportions of algal reads to the total eukaryotic reads ranged from 0.75 to 85.12%. The algal OTU richness in each sample ranged from 17 OTUs at station DF3 to 126 OTUs at station FN1 with the proportions of 7.86–26.47% (Figure 2B).

### Community structure of eukaryotic algae

The eukaryotic algae detected in this study included 131 genera in 31 classes of 9 phyla, i.e., Bacillariophyceae, Coscinodiscophyceae and Mediophyceae in Bacillariophyta, Chlorodendrophyceae, Chlorophyceae, Chloropicophyceae, Mamiellophyceae, Nephroselmidophyceae, Pedinophyceae, Pyramimonadophyceae, Trebouxiophyceae, and Ulvophyceae in Chlorophyta, Cryptophyceae in Cryptophyta, Dinophyceae, Ellobiophyceae, Noctilucophyceae, Perkinsea and Syndiniophyceae in Dinophyta, Coccolithophyceae in Haptophyta, Katablepharidophyceae in Katablepharidophyta, Bolidophyceae, Chrysophyceae, Dictyochophyceae, Eustigmatophyceae, Pelagophyceae, Raphidophyceae, Synurophyceae and Xanthophyceae in Ochrophyta, Prasinodermatophyceae in Prasinodermatophyta, and Florideophyceae and Rhodellophyceae in Rhodophyta.

Among the 454 algal OTUs, 201 OTUs (44.27%) were identified at the species level, 315 OTUs (69.38%) at the genus level, and the other 139 OTUs (30.62%) only at the class level after BLAST against the Silva and the NCBI databases. Sometimes more than one or two OTUs were annotated to the same taxa. Most of these OTUs were annotated to dinoflagellate species with the maximum of 16 OTUs to *Protoperidinium leonis*. Altogether 149 species were detected in this study, including 37 Bacillariophyta (39 OTUs), 39 Chlorophyta (40 OTUs), 2 Cryptophyta (2 OTUs), 49 Dinophyta (98 OTUs), 3 Haptophyta (3 OTUs), 1 Katablepharidophyta (1 OTU), 16 Ochrophyta (16 OTUs), and 2 Rhodophyta (2 OTUs) species (Supplementary Table 1). There were 53, 76, 95, and 91 species recorded in samples from Dafeng Port (DF), Xiangshan Bay (XS), Funing Bay (FN), and Dongshan Bay (DS), respectively. Most of the species or genera identified in surface sediments in this study were marine species, some were brackish or marine/freshwater species, and terrestrial and benthic species were also detected. Fifty-nine taxa have been reported to form resting stages (Supplementary Table 1).

**FIGURE 3:**
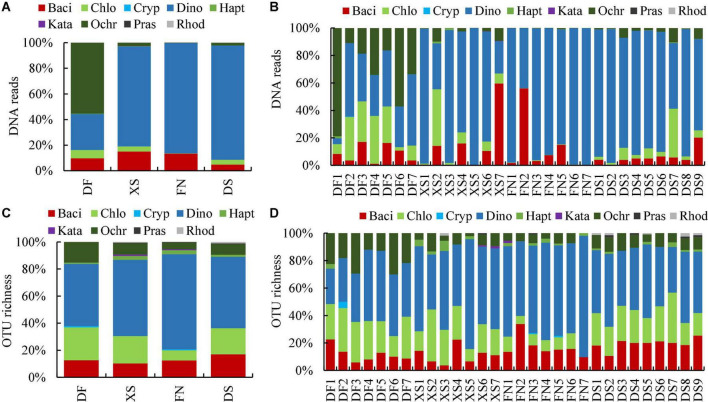
Relative abundance of DNA reads **(A,B)** and OTU richness **(C,D)** of benthic eukaryotic algae in surface sediments from the four sea areas at phylum level. DF, Dafeng Port; XS, Xiangshan Bay; FN, Funing Bay; DS, Dongshan Bay. Stations DF1–DF7 are in Dafeng Port, stations XS1–XS7 in Xingshan Bay, stations FN1–FN7 in Funing Bay, and stations DS1–DS9 in Dongshan Bay. Phyla are represented by their first four characters. Baci, Bacillariophyta; Chlo, Chlorophyta; Cryp, Cryptophyta; Dino, Dinophyta; Hapt, Haptophyta; Kata, Katablepharidophyta; Ochr, Ochrophyta; Pras, Prasinodermatophyta; Rhod, Rhodophyta.

Eukaryotic algal community was similar in Xiangshan Bay (XS), Funing Bay (FN), and Dongshan Bay (DS) in the East China Sea based on DNA reads at the phylum level (Figure 3A), which was predominated by dinophytes (dinoflagellates) with proportions to the algal reads of 78.14, 86.20, and 89.28%, respectively, followed by bacillariophytes (diatoms) with proportions of 15.01, 13.39, and 4.81%, respectively. However, algal community was different in Dafeng Port (DF) in the Yellow Sea, which was dominated by ochrophytes (55.47%), followed by dinoflagellates (28.07%) and diatoms (9.93%). The dominance of ochrophytes in Dafeng Port was caused by their predominance at stations DF1 and DF6, in which the relative proportions of ochrophytes to eukaryotic algae were 78.9 and 58.2%, respectively (Figure 3B). Chrysophyceae was the major class in Ochrophyta, contributing 85.13% to the ochrophyte reads. Therefore, algal community in DF was actually dominated by chrysophytes. Chlorophytes accounted for a certain proportion to eukaryotic algal community in the sediments, ranging from 0.22 (FN) to 6.52% (DF) (Figure 3A). The eukaryotic algal community structure differed among samples even in the same sea area, for example, dinoflagellates predominated at stations XS1 and XS3–XS5, while chlorophytes dominated at station XS2 and diatoms dominated at station XS7 (Figure 3B). Similarly, the eukaryotic algal community structures at stations FN2 and DS7 were different from those of other samples in the same sea area.

The OTU richness of eukaryotic algae identified in each sea area was 111, 204, 265, and 201 OTUs in DF, XS, FN, and DS, respectively. The OTU richness identified in each sample ranged from 17 to 126 OTUs, with an average of 62 OTUs. The composition of eukaryotic algal OTU richness was similar, which was dominated by dinoflagellates (Figure 3C), with the relative OTU richness of 45.95, 56.37, 70.19, and 52.74% in DF, XS, FN and DS, respectively. On the other hand, chlorophytes (7.55–20.32%), diatoms (10.29–16.92%), and ochrophytes (4.91–15.32%) accounted for a certain proportion in OTU richness. The profile of OTU richness in each sample was similar, which was co-dominated by dinoflagellates, chlorophytes and diatoms successively (Figure 3D).

**FIGURE 4:**
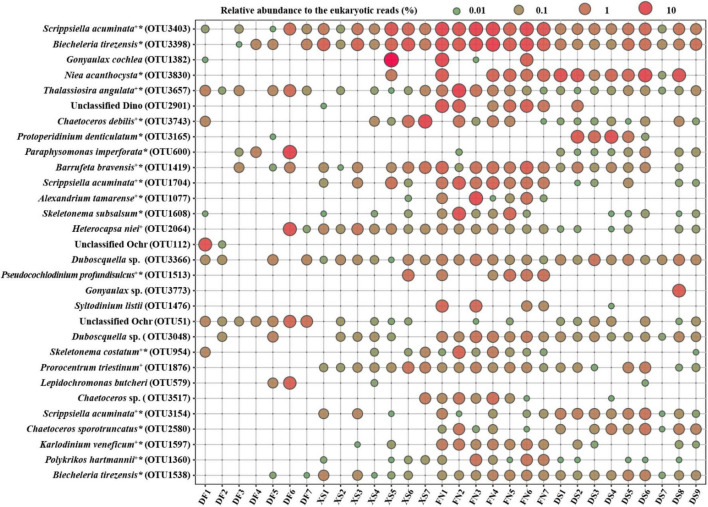
The bubble chart showing DNA reads of the top 30 abundant OTUs in each station. +: HAB species; *taxa reported to form resting stages. Supporting evidence to HAB species and formation of resting stages are listed in Supplementary Table 1.

Figure 4 illustrates DNA reads of the top 30 abundant OTUs. Most of the top abundant OTUs belonged to dinoflagellates, except for 6 OTUs in diatoms, and 4 OTUs in Chrysophyceae. Most of the identified species with high abundance were those confirmed to form resting stages (Figure 4 and Supplementary Table 1), while those identified at the genus level were cyst/spore forming genera such as *Gonyaulax* and *Chaetoceros*, or the endoparasitic dinophytes such as *Duboscquella* spp. Furthermore, there were 13 OTUs of 11 HAB species within the top 30 abundant OTUs. No OTUs were present at all stations, and 12 OTUs occurred in all of the four sea areas. *Scrippsiella acuminata* was the first dominant species in this study, and 8 OTUs of *S. acuminata* were recorded in this study (Supplementary Table 1), three of which were the top one, 11th and 26th dominant OTUs, respectively (Figure 4).

**FIGURE 5:**
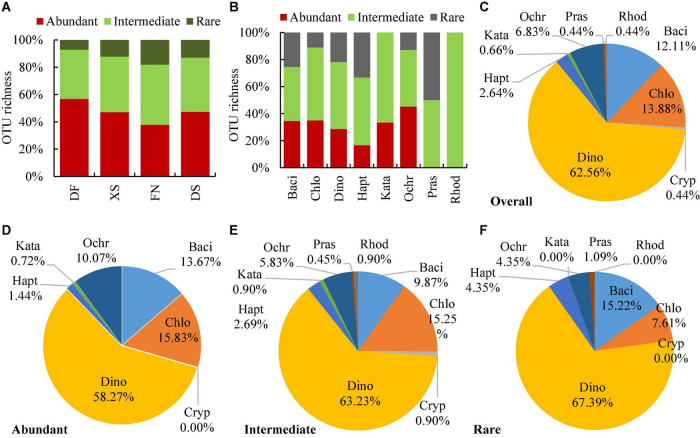
Relative OTU richness of overall, abundant, intermediate, and rare OTUs in the four sea areas **(A)** and each phylum of eukaryotic algae **(B–F)**. The abundant, intermediate, and rare OTUs are defined as those accounting at least 0.1, 0.01–0.1%, and less than 0.01% of the total eukaryotic reads of at least one sample, respectively. DF, Dafeng Port; XS, Xiangshan Bay; FN, Funing Bay; DS, Dongshan Bay. Phyla are represented by their first four characters as the same with Figure 3.

The abundant OTUs were supported by 0.1% of eukaryotic reads in at least one sample, the intermediate and rare OTUs were supported by <0.1% but ≥0.01% of reads, and by <0.01% of reads, respectively. There were 139 abundant, 223 intermediate, and 92 rare OTUs within the 454 algal OTUs. Algal OTUs were dominated by abundant and intermediate OTUs in all four sea areas (Figure 5A), with the percentages of 37.74–56.76 and 36.04–44.05%, respectively. Intermediate OTUs dominated in almost all algal phyla, accounting for 40–100% of the algal OUTs (Figure 5B). While abundant and rare OTUs accounted for 0–45.19 and 0–50%, respectively. However, no matter in overall algal OTUs (Figure 5C) or OTUs with different abundance (Figures 5D–F), dinoflagellates contributed the majority of OTU richness, which contributed 62.56, 58.27, 63.23, and 67.39% to the overall, abundant, intermediate, and rare OTUs, respectively. Chlorophytes, diatoms, and ochrophytes ranked the second to the fourth abundance in OTU richness, and chlorophytes contributed more OTUs to the abundant and intermediate OTUs, more diatoms to the rare and abundant OTUs, and more ochrophytes to the abundant OTUs. Among 149 eukaryotic algae identified at the species level, abundant species dominated as well, and the abundant, intermediate and rare species were 77, 55, and 17 species, respectively (Supplementary Table 1).

### Alpha and beta diversity indexes of eukaryotic algae

**FIGURE 6:**
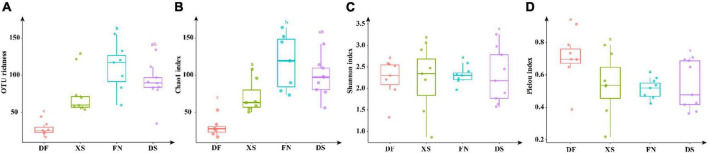
Alpha diversity indexes of eukaryotic algae in the four sea areas. The significant difference of alpha indexes between sea areas was performed by TukeyHSD test in R4.1.0. Data bars carrying different letter designations (a–c) indicated significant difference (*p* < 0.05, TukeyHSD test), and the same and similar letter designations (e.g., a and ab, b and ab) denoted no significant difference (*p* > 0.05). **(A)** OTU richness, **(B)** Chao 1, **(C)** Shannon, **(D)** Pielou.

Operational Taxonomic Unit richness and Chao1 index were significantly lower in DF than the other three sea areas (*p* < 0.01), and high values were obtained in FN and DS (Figure 6). However, values of the Shannon index were comparable among the four sea areas (*p* > 0.05), and the highest value of Pielou evenness index was recorded in DF among the four sea areas though no significant difference among the four sea areas.

**FIGURE 7:**
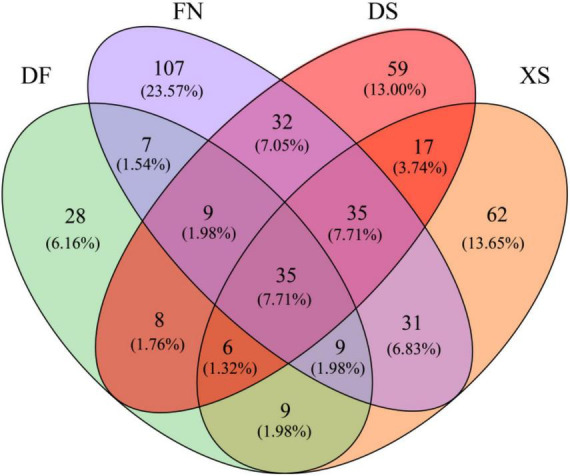
Venn diagram highlighting the degree of overlap of algal OTUs among the four sea areas. DF, Dafeng Port; XS, Xiangshan Bay; FN, Funing Bay; DS, Dongshan Bay.

The Venn diagram highlighted the differences of algal communities among the four sea areas (Figure 7). Only 35 OTUs shared among the four sea areas. Particularly, FN had the highest OTU richness, and 107 unique OTUs were detected. The numbers of unique OTUs were comparable in DS and XS, which were 59 and 62 OTUs, respectively. Dafeng Port (DF) had the lowest OTU richness and only 28 unique OTUs occurred in DF.

**FIGURE 8:**
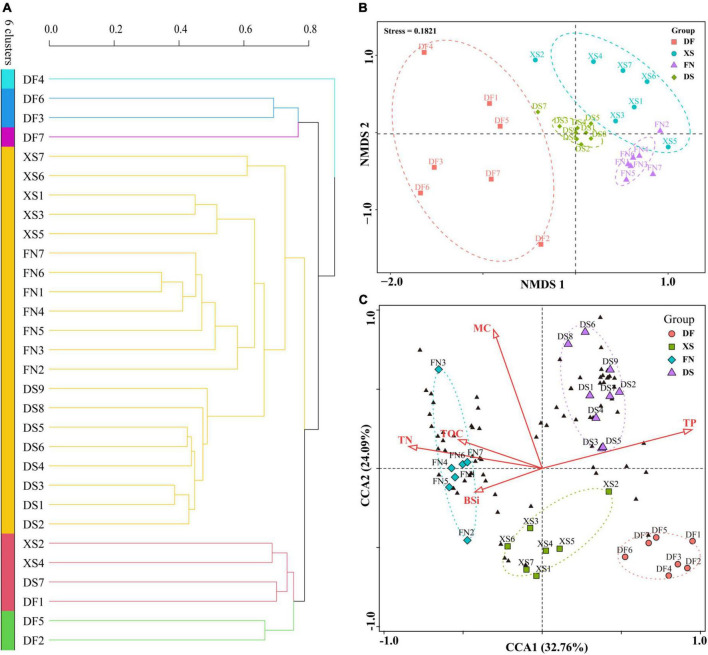
Cluster, non-metric multidimensional scaling (NMDS), and canonical correspondence analysis (CCA) of the thirty sediment samples from the four sea areas along the southeast Chinese coast based on the eukaryotic algal community. Cluster and NMDS analyses were constructed based on the Bray-Curtis (BC) index. CCA was conducted based on biogenic elements and eukaryotic algal OTUs. **(A)** Cluster analysis. **(B)** NMDS analysis. **(C)** CCA plot ordination sampling stations, algal OTUs (black triangles), and biogenic elements (red arrows), DF, Dafeng Port; XS, Xiangshan Bay; FN, Funing Bay; DS, Dongshan Bay.

The thirty samples were clustered into six groups (Figure 8A), in which samples from DF were separated from the other samples. Five samples from DF were clustered into a red group, while the other two samples (DF2 and DF4) were ungrouped, and then grouped together with the red group. Most samples from the other three sea areas, including nine samples from DS, six from XS, and two from FN, were clustered into a large light blue group. Station FN1 was ungrouped, and the other four FN samples were grouped together with station XS6 into a purple group.

Non-metric multidimensional scaling analysis well differentiated samples in the four sea areas, and samples in the same sea area were grouped together (Figure 8B). Samples from DF scatteredly distributed at the negative axis of NMDS1 without intersection with samples from the other sea areas. In addition, most samples from XS scattered in the positive axis of NMDS1 and NMDS2. Samples from FN and DS distributed more concentratedly. The nine samples from DS were clustered in a small area near the origin. While samples from FN mostly located in the fourth quadrant, which had a small intersection with the XS group.

### Canonical correspondence analysis between algal community and biogenic elements

Canonical correspondence analysis was conducted based on eukaryotic algal community and biogenic elements (Figure 8C). CCA1 and CCA2 explained 32.76 and 24.09% of the environmental and biological variables, respectively. TP had a positive contribution to axis CCA1, while other biogenic elements had negative contributions to CCA1. Most of the biogenic elements had positive contributions to CCA2 except for a weak negative contribution of BSi. Algal OTUs were mostly clustered into two groups, one of which scattered in the negative direction of CCA1, which had small angles with TOC, TN, and BSi, indicating that the distribution of these algal OTUs was affected by TOC, TN, and BSi. The other group distributed in the first quadrant with acute angles with TP and MC, indicating that they were influenced by TP and MC. Samples in the four sea areas were separately grouped, which showed different environmental characteristics and eukaryotic algal community structure in different sea areas. Nine samples from DS were clustered in the first quadrant, indicating higher TP and MC but lower contents of other elements in DS. Seven samples from FN were grouped in the negative direction of CCA1, indicating higher TN, TOC, and BSi contents in FN. The XS samples were mostly located along the negative axis of CCA2, indicating the high BSi concentration. DF samples were located in the lower right side of the fourth quadrant, indicating the high TP content and low concentrations of other elements. Meanwhile, algal OTUs were mostly distributed within the DS and FN groups, indicating the abundant OTU richness in the two sea areas as shown in Figure 6A.

### Distribution of harmful algal bloom species

**FIGURE 9:**
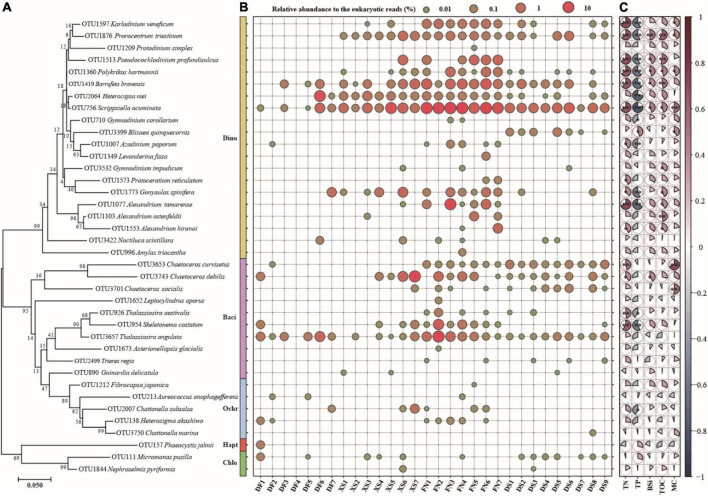
The phylogenetic analysis (**A**, left), distribution (**B**, middle) of the harmful algal bloom (HAB) species and pairwise comparison of environmental factors and HAB species (**C**, right). The phylogenetic tree was constructed using the Maximum Likelihood (ML) method with 1000 bootstrap replicates. Blue in the pairwise comparison **(C)** represents negative correlation, red represents positive correlation, and pie area represents correlation value. The pies with * indicate *p* < 0.05 and the pies with ^**^ indicate *p* < 0.01.

Thirty-eight potentially toxic/harmful and/or bloom species were detected in this study (Supplementary Table 1), including 20 taxa in Dinophyta (dinoflagellates), 10 in Bacillariophyta (diatoms), 5 in Ochrophyta, 2 in Chlorophyta, and 1 in Haptophyta (Supplementary Table 1 and Figure 9). There were 15, 20, 33, and 21 HAB taxa recorded in DF, XS, FN, and DS, respectively, and 9 HAB taxa were recorded in all of the four sea areas. Notably, 24 HAB species detected in this study were abundant species (Supplementary Table 1). Some of the HAB species occurred widely and dominantly in this study, such as *Scrippsiella acuminata*, *Thalassiosira angulata*, *Chaetoceros debilis*, *Barrufeta bravensis*, *Alexandrium tamarense*, *Heterocapsa niei*, *Pseudocochlodinium profundisulcus* (formerly *Polykrikos geminatus and Cochlodinium geminatum*), *Skeletonema costatum*, *Gonyaulax spinifera*, *Prorocentrum triestinum*, *Karlodinium veneficum*, and *Polykrikos hartmannii* (Figure 9B) with the average relative abundance to the eukaryotic reads of 0.17–5.13%. The maximum relative abundances were >10% (11.18–23.35%) for five HAB species (*S. acuminata*, *T*. *angulata*, *C*. *debilis*, *A*. *tamarense*, and *H*. *niei*), and 1–10% (1.23–5.47%) for seven HAB species (*B*. *bravensis*, *S. costatum*, *P*. *profundisulcus*, *G. spinifera*, *P*. *hartmannii*, *Alexandrium hiranoi*, and *P*. *triestinum*).

In addition, 27 HAB species have been reported to form resting stages (Supplementary Table 1). Although some HAB species have not been reported to form resting stages, such as *Gonyaulax cochlea*, *H. niei*, and *Amylax triacantha*, their widespread occurrence in sediments suggests that they may be resting stage-forming species, however without laboratory evidences. Some HAB species produce toxins, which have potential risks to human health and marine animals (Supplementary Table 1), including the PSP producers *Alexandrium ostenfeldii* and *A*. *tamarense*, the goniodomine A producer *A. hiranoi*, the azaspiracid shellfish poisoning (AZP) producer *Azadinium poporum*, the yessotoxin (YTX) producers *G. spinifera* and *Protoceratium reticulatum*, the karlotoxin producer *K. veneficum*, the ichthyotoxic species *P. profundisulcus*, *P. hartmannii*, *Aureococcus anophagefferens*, *Chattonella marina*, *Chattonella subsalsa*, *Fibrocapsa japonica*, and *Heterosigma akashiwo*.

Harmful algal blooms species occurred abundantly and widely in FN (Figure 9B). Eight HAB species (*S. acuminata*, *T. angulata*, *B. bravensis*, *S. costatum*, *K. veneficum*, *P. triestinum*, *H. niei*, and *Chaetoceros curvisetus*) occurred in all samples from FN with average relative abundance of 0.04–17.10% to the eukaryotic reads (Figure 9B). *S. acuminata* was the most abundant HAB species in this study and in FN, with the relative abundance of 5.35–23.34% and average of 17.10% to the eukaryotic reads in FN. The PSP producer *A. tamarense* was detected in six samples from FN with the maximum relative abundance of 13.11% in FN3. The YTX producer *G. spinifera*, karlotoxins species *K. veneficum*, and ichthyotoxic species *P. profundisulcus* and *P. hartmannii* occurred abundantly and widely in FN as well with the average abundances of 0.24–0.83% to the eukaryotic reads (Figure 9B). The relative abundances of HAB species were high in XS as well, including *C. debilis* with the average relative abundance of 3.04% to the eukaryotic reads, *S. acuminata* (2.83%), *B. bravensis* (0.50%), *G. spinifera* (0.36%), *P. triestinum* (0.32%), *H. niei* (0.30%), and *P. profundisulcus* (0.24%). There were 21 HAB species recorded in DS, and the dominant HAB species included *S. acuminata* (the average relative abundance of 1.34% to the eukaryotic reads), *P. triestinum* (0.17%), *B. bravensis* (0.16%), and *C. curvisetus* (0.10%). Only 15 HAB species were recorded in DF, and the dominant HAB species included *H. niei* (the average relative abundance of 1.60% to the eukaryotic reads), *T. angulata* (0.49%), *S. acuminata* (0.33%), and *G. spinifera* (0.14%).

A phylogenetic tree clustered the 38 HAB species in five phyla (Figure 9A), in which 20 dinoflagellate species were grouped into a large yellow clade, diatoms (purple) and ochrophytes (blue) were clustered together, and one haptophyte (red) and two chlorophyte species (green) were grouped together. The relative abundance of most HAB species was positively correlated with TN, BSi, TOC, and MC, however negatively correlated with TP (Figure 9C). Fifteen HAB species showed significant positive correlations with TN (*p* < 0.05, or <0.01), and 6–8 HAB species were significantly positively correlated with BSi, TOC, and MC (*p* < 0.05, or <0.01). No HAB species displaced significant negative correlations with TN, BSi, TOC, and MC. The relative abundance of 14 HAB taxa had significant negative correlations with TP (*p* < 0.05, or <0.01), and only one species (*Blixaea quinquecornis*) showed significant positive correlation with TP (*p* < 0.05). The distribution of many abundant HAB species, such as *S. acuminata*, *B. bravensis*, *P. triestinum, P. profundisulcus*, and *P. hartmannii*, showed significant positive correlations with TN, BSi, and TOC, while negative correlations with TP. Other abundant HAB species, e.g., *H. niei*, *K. veneficum*, *G. spinifera*, *A. tamarense*, *C. debilis*, and *S. costatum*, showed similar correlations with biogenic elements, but not significantly in some cases.

## Discussion

### Composition of eukaryotic algae in surface sediments from the four sea areas

A total of 3,899 eukaryotic OTUs including 454 algal OTUs were detected in the 30 surface sediment samples from the four sea areas along the southeast Chinese coasts in this study. The OTU richness in this study was much higher compared with those observed in surface sediments from other sea areas, for example 180 OTUs of microalgal species were identified from 13 stations across the Black Sea (Dzhembekova et al., 2018), and 218 algal OTUs in 16 stations along the southern Chinese coast (Liu et al., 2020a). The results suggest that investigations in diverse sea areas may enhance the OTU richness. Actually, OTU richness obtained in each sea area (111–265 OTUs) was comparable to those reported in other studies. However, diversity of eukaryotic algae in sediments is generally lower than those of phytoplankton in water column. Chen et al. (2021) detected 4,689 OTUs of phytoplankton in four cruises in the Jiaozhou Bay, the Yellow Sea, China. Penna et al. (2017) detected 1,398 OTUs of autotroph plankton from 20 plankton samples collected at 19 sampling sites across the coastal areas of the Mediterranean Sea. Most phytoplankton vegetative cells are generally not able to survive in sediments for a long time, and only resting stages of phytoplankton are well preserved in sediments after long-term cold-dark storage (reviewed by Ellegaard and Ribeiro, 2018). In the present study, the sediment samples were stored in a dark place at 4–8°C for 6 months before DNA extraction to ensure that the algal DNA extracted from sediments was mostly from resting stages (Piredda et al., 2017). Therefore, the algal OTU richness in sediments is generally lower than that obtained from the water column, because only a small component of phytoplankton could form resting stages (Head, 1996; McQuoid and Hobson, 1996).

Fifty-nine of the 149 taxa identified at the species level in this study have been reported to form resting stages (Supplementary Table 1). The ability of forming resting stages by other taxa remains unclear. For example, *Gonyaulax cochlea* occurred abundantly in sediments (Supplementary Table 1 and Figure 4), suggesting that it is a cyst-forming species. Meanwhile, cyst formation is an important characteristic in genus *Gonyaulax* (Matsuoka and Fukuyo, 2000), and maybe also in this species. Those that do not produce resting stages were generally detected with low numbers of DNA reads, which might also represent fragments of vegetative cells or even DNA relics because the dark cold treatment before DNA extraction could not exclude the fragmental DNA released from the dead cells. These DNA fragments should generally be represented as rare OTUs (Shang et al., 2019), which would not influence the relative abundance of phytoplankton resting stages and the benthic algal community significantly. Meanwhile, whole-genome amplification was applied in this study, and rare OTUs might be screened from the dominant OTUs in the PCR process. As singletons and doubletons were removed and DNA reads were normalized to the fewest reads in this study, the very rare OTUs were removed after normalization, which might exclude majority of DNA fragments. Actually, abundant and intermediate OTUs dominated in the algal OTUs (Figure 5), and also in those identified to the species level (Supplementary Table 1). The rare OTUs detected in this study suggested their presence in the studying sea areas in the form of resting stages and/or vegetative cells. It has been suggested that the rare taxa may include key species regulating the functioning of aquatic environments (Debroas et al., 2015; Lynch and Neufeld, 2015). Furthermore, the rare taxa can act as seeds for seasonal succession or sporadic blooms (Logares et al., 2014).

Dinoflagellate sequences dominated in the benthic algal reads in this study. Dinoflagellates also had the highest OTU richness. The algal community structure in this study is comparable to those reported in sediments from the other coastal sea areas based on metabarcoding analysis, in which dinoflagellate DNA reads and OTU richness were predominated (Dzhembekova et al., 2018; Liu et al., 2020a). Dinoflagellates have much higher DNA copies (up to thousands) than the other protists (dozens to hundreds) (Lin, 2011), which may lead to their over-representation in metabarcoding studies. In addition, dinoflagellate cysts constitute an important component of sedimentary assemblages of microalgal resting stages (Ellegaard and Ribeiro, 2018). Actually, most dominant dinoflagellates detected at the species level in this study were cyst-forming taxa (Figure 4).

Though diatoms predominated in phytoplankton group in the four sea areas (Chen et al., 2004; Dong, 2012; Quan et al., 2015; Wu, 2021), diatom DNA reads contributed only 4.18–15.01% to the eukaryotic algae in the present study (Figure 3A). Resting spores have been reported only in some centric diatoms (McQuoid and Hobson, 1996). Diatom OTUs obtained from sediment metabarcoding are mostly a few resting stages forming species, such as species in genera *Chaetoceros*, *Skeletonema*, and *Thalassiosira* (Montresor et al., 2013; Piredda et al., 2017; Dzhembekova et al., 2018). Therefore, the diversity and abundance of diatoms are usually low in sediment metabarcoding studies.

### Benthic algal communities in the four sea areas

The eukaryotic algal community structure in Dafeng Port (DF) of the Yellow Sea was obviously different from those in the other three sea areas. Specifically, chrysophytes dominated in DF, while dinoflagellates predominated in the other sea areas. Meanwhile, DNA reads, OTU richness and Chao1 index of eukaryotic algae in DF were also significantly lower. Results from the cluster, NMDS, and CCA analyses showed that samples from DF were clustered apart from the other three sea areas (Figure 8). Differences in phytoplankton resting stages abundance and assemblage composition between areas are primarily caused by two factors: differences in the abundance of vegetative cells and in their resting stage production efficiencies, and/or differences in the sedimentary regime (Balch et al., 1983; Joyce et al., 2005). DF is an open sea area with well exchange with the outside sea water. The sediments in DF are mostly composed by coarse particles such as silty sand and sandy-silty sand (Zhang et al., 2016). It is well known that phytoplankton resting stages such as dinoflagellate cysts behave like fine particles (Dale, 1983). Therefore, the coarse sediment in DF is not beneficial for the deposition of resting stages (Yamaguchi et al., 1995). Furthermore, DF had the lowest TOC, TN, and BSi within the four sea areas (Supplementary Table 2), indicating the low nutrient level and primary production in DF. In addition, DF is a large port and also an important offshore wind farm (Ling, 2014). Intensive human activities, such as offshore engineering construction and shipping, have caused severe sediment disturbance and increased terrigenous sediment input. All of these resulted in a decrease abundance and richness of eukaryotic algae in sediments in DF.

The other three sea areas are located in the East China Sea, and are semi-closed bays with less exchange with the outside sea waters (Figure 1), and also with rich nutrients and high phytoplankton biomasses due to the high input of nutrients from aquaculture activities (Chen et al., 2004; Wu et al., 2017; Wu, 2021). Therefore, most of the phytoplankton resting stages in these three bays are deposited locally due to less exchanges, resulting in rich eukaryotic algae in sediments. Furthermore, long-term aquaculture activities have accumulated a large amount of organic-rich sediment with high biogenic elements (Supplementary Table 2), which is favorable for the deposition of phytoplankton resting stages.

Sediment samples from different sea areas were collected in different years and seasons in this study, which might limit the information obtained from the comparison of eukaryotic algal community in sediments from different coastal areas. It is well-known that phytoplankton community structure changes rapidly, and can vary greatly depending on sampling time (Li et al., 2022). However, phytoplankton resting stages in sediments were assemblages of those formed during a period of time and can remain viable in sediments from years up to a century depending on the species (McQuoid, 2002; Lundholm et al., 2011), thereby providing an integrated record over time of the presence of resting stage producing species (Joyce et al., 2005). The upper sediments (0–2 cm) were analyzed in this study due to the high germination potential of resting stages in the top sediments when they are suspended to the water column with favorable temperature and oxygen availability (Anderson et al., 2014; Brosnahan et al., 2020; Rodríguez-Villegas et al., 2021). The sediment accumulation rates are generally between 0.2–1.0 cm/year in the coastal sea areas without inputs of large rivers, such as 0.12–0.26 cm/year in the southern Yellow Sea (Li, 2015; Liu, 2015), 0.32–0.65 cm/year in Xiangshan Bay (Sun W. et al., 2017; Liang, 2019), and 0.46–0.69 cm/year in coastal sea areas along Zhejiang and Fujian provinces of China (Li, 2015; Liu, 2015). Therefore, the upper 2 cm sediments hence represent a time interval of 3–8 years. Despite of a sampling time span of 5 years (2014–2018), the assemblages of resting stages in surface sediments in our study indicated the occurrence and distribution of HAB species in the southeast coast of China in recent years (ca. 2010–2018) and revealed the potential risk of HABs in the future years and even decades. Due to the difficulties in sediment collection, using sediment samples collected at different time to map the distribution of HAB species has been reported by other authors as well (Tang et al., 2019; Hu et al., 2021).

### Potential risks of harmful algal blooms

Resting stage is a specific dormant phase in the life cycle of many phytoplankton taxa. Though only a small part of phytoplankton species can form resting stages (Head, 1996), a lot of HAB species have the ability to form resting stages (Bravo and Figueroa, 2014). Resting stages can germinate into vegetative cells under favorable conditions, which provides an inoculum for blooms (Joyce et al., 2005; Olli and Trunov, 2010; Anderson et al., 2014; Sildevera et al., 2017). In turn, under unfavorable conditions, vegetative cells will form resting stages, which can remove substantial numbers of cells from blooms as they terminate and also seed for the next bloom (Stock et al., 2007; Olli and Trunov, 2010; Anderson et al., 2014). The distribution, composition, and abundance of HAB species can be obtained by mapping the resting stages in surface sediments (Figueroa et al., 2018), which provide valuable information regarding previous and future blooms (Bravo and Figueroa, 2014; Sildevera et al., 2017; Rodríguez-Villegas et al., 2020, 2021). Therefore, surveys of resting stages in surface sediment may serve to provide effective early warning of toxic algal blooms in a specific region (Bravo et al., 2006).

Thirty-eight HAB species were detected in our study, including those producing toxins penitently harmful to human health. Most HAB species found in this study can form resting stages (27 to 38 species, Supplementary Table 1), and 24 HAB species belonged to abundant species and occurred widely and abundantly in the sediments (Supplementary Table 1 and Figure 9B). Notably 33 HAB species occurred in sediment samples from Funing Bay. The result suggested high potential risks of HABs in the southeast Chinese coastal areas, especially in FN. Funing Bay is an important aquaculture area for fish, shrimp, crab and shellfish cultivation, and the abundant occurrence of these HAB species suggests the potential risk of the accumulation of toxins in shellfish and/or the massive death of farmed fish by ichthyotoxic blooms. The distribution of most abundant HAB species showed positive correlations with TN, BSi, and TOC (Figure 9C), suggesting that organic matters originated from aquaculture activities and consequent high diatom productivity may have a significant influence on the distribution of HAB species and facilitate the occurrence of HABs.

Dinoflagellates account for 75% of all HAB species (Smayda, 1997), and they were also the most important HAB group identified in this study. Many species in genus *Alexandrium* are responsible for paralytic shellfish poisoning (PSP). *Alexandrium* blooms have been frequently recorded in the southeast Chinese coastal waters in the recent two decades (Gu et al., 2022). Meanwhile, cysts of *Alexandrium* are widely distributed in sediments form Chinese coasts (Tang et al., 2021). PSP toxins have been detected in both phytoplankton and shellfish samples from Funing Bay and Dongshan Bay, and human poisoning events have been reported as well after eating contaminated shellfish (Guo, 2012; Chen et al., 2018; Xie, 2018; Wang, 2020). Four *Alexandrium* species were detected in this study, i.e., *A. tamarense*, *A. hiranoi*, *A. ostenfeldii*, and *A. satoanum*, among which *A. tamarense* and *A. ostenfeldii* are PSP producers (Lundholm et al., 2009 onward). *A. tamarense* widely and abundantly occurred in FN with the maximum relative abundance of 16.11% to the eukaryotic reads. However, none of PSP producers were detected in sediments from DS (Supplementary Table 1), perhaps because of the low vegetative cells in the water column (Wang, 2020), which were not enough to form resting stages to be detected in metabarcoding. The high relative abundance of *Alexandrium* spp. detected in sediments from FN in our study suggested high potential risk of *Alexandrium* blooms and PSP events in Funing Bay. In fact, a mixed blooms of *Noctiluca scintillans* and *A. tamarense* occurred in Funing Bay in April 2002 (Liang et al., 2019). Monitoring data between 2013 and 2015 showed that PSP toxins in shellfish from Funing Bay were contaminated to a certain degree, and about 20–30% of shellfish samples were above the national standard level (4 Mu/g) (Liang et al., 2019).

*Gonyaulax spinifera* and *Protoceratium reticulatum* are the potential YTX producers (Chikwililwa et al., 2019). *G. spinifera* distributed in all of the four sea areas, and abundantly and widely occurred in XS and FN (Figure 9B). *P. reticulatum* was detected only in FN in low abundance. As a cyst-forming species, *G*. *spinifera* can form variety types of cysts, mostly belonging to the cyst genus *Spiniferites* with wide morphological variations (Rochon et al., 2009), and its cysts were widely recorded in sediments (Tang et al., 2021). YTX toxins were detected in phytoplankton and shellfish samples from the Chinese coasts (Jiang et al., 2017; Liu et al., 2021; Zheng et al., 2021). High YTX and Homo YTX levels were detected in shellfish samples from Funing Bay during surveys between 2017 and 2020 (Zheng et al., 2021). The high relative DNA reads of *G. spinifera* in sediments from XS and FN in this study also indicate certain risks of YTX toxin in the two sea areas.

*Karlodinium veneficum* is a small sized naked dinoflagellate, which produces a group of polyketide toxins (karlotoxins) exerting hemolytic, ichthyotoxic and cytotoxic effects on fishes and aquatic animals (Place et al., 2012). However, *K. veneficum* had been greatly overlooked in the routine phytoplankton survey due to the small size, fragile in fixation samples and morphological similarity to other unarmored species (Huang et al., 2019). Since the first bloom of *K*. *veneficum* reported in the South China Sea in 2003, its blooms occurred in the Chinese coastal sea areas frequently (Liu et al., 2020b; Gu et al., 2022). Cysts of this species were detected widely in sediments from the China Sea as well (Liu et al., 2020b). *K. veneficum* occurred in three sea areas in this study, and was recorded in all of seven stations of Funing Bay with relative reads of 0.18–0.85% to the eukaryotic reads (Figure 9B). Considering the association of its blooms and massive fish killings (Place et al., 2012), the wide distribution of *K*. *veneficum* in Funing Bay is worthy of attention.

Many species of *Azadinium* are responsible for AZP (Tillmann et al., 2014). Two species of *Azadinium* (*A. poporum* and *A. trinitatum*) were detected in this study, and *A*. *poporum* has the potential to produce AZP (Krock et al., 2012). *A*. *poporum* was reported to distribute widely in surface sediments from the Chinese coasts (Gu et al., 2013; Liu et al., 2020a), and 13 out of 16 strains of *A*. *poporum* from different geographic locations along the Chinese coastline contained AZP (Krock et al., 2014). The relative abundance of *A*. *poporum* was generally low however up to 0.14% of the eukaryotic reads in FN3 (Figure 9B). On the other hand, the non-toxic *A*. *trinitatum* distributed in XS, FN, and DS (Supplementary Table 1). Cyst formation is an important characteristic in genus *Azadinium* (Tillmann et al., 2016). The wide distribution of *Azadinium* along the Chinese coasts (this study, Gu et al., 2013; Luo et al., 2013; Liu et al., 2020a) and worldwide in sea areas (Akselman and Negri, 2012; Tillmann et al., 2014) indicates a global distribution of this genus (Tillmann et al., 2014).

The ichthyotoxic species, whose blooms have been associated with massive fish kills, included the dinoflagellates *Pseudocochlodinium profundisulcus* and *Polykrikos hartmannii*, the pelagophyte *Aureococcus anophagefferens*, and the raphidophytes *Chattonella marina*, *C. subsalsa*, *Fibrocapsa japonica*, and *Heterosigma akashiwo* (Supplementary Table 1). *P. profundisulcus* is a common bloom species in the Pearl River Estuary of the South China Sea, and its blooms have frequently occurred since 2006 (Ou et al., 2010; Shen et al., 2012). *P. profundisulcus* was initially identified as *Cochlodinium geminatum* (Ou et al., 2010) and subsequently reclassified as *Polykrikos geminatus* (Qiu et al., 2013). Recently, based on morphological comparisons and phylogenetic analyses for the SSU and partial LSU rRNA genes, Hu et al. (2021) has re-described it as a new species of a new genus, *Pseudocochlodinium profundisulcus* gen. et sp. nov. Moreover, metabarcoding investigation of surface sediment samples and LM observation of cyst morphology demonstrated a wide distribution along the entire Chinese coast, with the highest abundance observed in the SCS (Hu et al., 2021). *P. profundisulcus* was detected in sediment samples from five stations in FN and one station in XS, and ranked the top 17th abundant OTU with the maximum relative abundance of 1.91% to the eukaryotic reads (Figure 4), suggesting its abundant occurrence in the East China Sea as well. *P. hartmannii* occurred in three sea areas except for DF (Supplementary Table 1 and Figure 9B). Though its bloom has been only reported to occur in the Forge River Estuary, NY, United States (Tang et al., 2013), cysts of these species are widely distributed in the worldwide sea areas (Fujii and Matsuoka, 2006; Tang et al., 2021). Other ichthyotoxic species occurred sporadically in low abundance in this study.

*Scrippsiella acuminata* is a cosmopolitan dinoflagellate species in coastal waters (Wang et al., 2007). Blooms of this species were reported in sea areas worldwide, and sometimes occurred successively with diatom blooms (Wang et al., 2007; Zinssmeister et al., 2011; Tian et al., 2021). *S. acuminata* easily forms resting cysts in both natural sea waters and laboratory conditions, and cysts play an important role in its recurrent blooms (Wang et al., 2007). Previous studies showed that cysts of *S. acuminata* were widely and abundantly distributed in sediments from all Chinese coastal sea areas (Tang et al., 2021). *S. acuminata* was the most abundant eukaryotic alga in this study, and occurred in almost all stations due to its prevalent distribution and easy cysts formation.

*Barrufeta bravensis* is a new species in class Gymnodiniales, which formed a bloom in the northwestern Mediterranean Sea (Sampedro et al., 2011). This species has not been reported in China yet. However, it was widely and abundantly distributed in this study, indicating that it is a common species in the southeast Chinese coasts. *Heterocapsa niei* is a small dinoflagellate, which sometimes co-occurred with other HAB species and formed harmful blooms (Reifel et al., 2002; Crespo et al., 2008). *H. niei* occurred abundantly in this study, and ranked the top 14th. Although there are no evidences of these bloom causative dinoflagellates (*S. acuminata*, *B. bravensis*, and *H. niei*) to produce toxins, their massive blooms have caused damages to the ecological environments and economic losses to mariculture (Reifel et al., 2002; Wang et al., 2007; Crespo et al., 2008).

## Conclusion

This study enriched our understanding of eukaryotic algae in surface sediments from the four sea areas along the southeast Chinese coastal waters. Diverse eukaryotic algae were recorded, including 454 OTUs belonged to 131 genera in 31 classes of 9 phyla. Among 149 taxa identified at the species level, 59 taxa have been reported to form resting stages. The eukaryotic algal community structure was quite different in Dafeng Port of the southern Yellow Sea. Though algal OTUs were dominated by abundant and intermediate OTUs, rare OTUs also played an important role in ecosystem stability. Thirty-eight potentially HAB species were detected in this study, including the PSP producers *Alexandrium ostenfeldii*, and *A. tamarense*, the goniodomine A producer *A. hiranoi*, the AZP producer *Azadinium poporum*, the YTX producers *Gonyaulax spinifera* and *Protoceratium reticulatum*, the karlotoxin producer *Karlodinium veneficum*, the ichthyotoxic species *Pseudocochlodinium profundisulcus*, *Polykrikos hartmannii*, *Aureococcus anophagefferens*, *Chattonella marina*, *C. subsalsa*, *Fibrocapsa japonica*, and *Heterosigma akashiwo*. Pairwise comparison between biogenic elements and HAB species suggests that eutrophication and consequent increase in diatom productivity may have a significant influence on the distribution of HAB species and facilitate the occurrence of HABs. In addition, 27 HAB species have been reported to form resting stages. Notably, HAB species occurred abundantly and widely in Funing Bay, suggesting the potential risk of the accumulation of toxins in shellfish and/or the massive death of farmed fish by ichthyotoxic blooms.

## Data availability statement

The datasets presented in this study can be found in online repositories. The names of the repository/repositories and accession number(s) can be found below: https://www.ncbi.nlm.nih.gov/genbank/, PRJNA837864.

## Author contributions

ZW designed the experiment and prepared the manuscript. LP prepared the manuscript. YT and LX designed the experiment. CX and WW completed the experiment. YZ and CX conducted the statistical analyses. YY prepared the manuscript. All authors contributed to the article and approved the submitted version.

## Footnotes

1
http://qiime.org

2
http://drive5.com/uparse/

3
www.arb-silva.de

4
https://www.ncbi.nlm.nih.gov/

5
https://www.ncbi.nlm.nih.gov/sra/

## Supplementary material

Supplementary Figure 1 Rarefaction curves of DNA reads based on OTUs at 97% sequence similarity.
