# Supplementary figures and images for: Metabarcoding of harmful algal bloom species in sediments from four coastal areas of the southeast China
Source: Front Microbiol. 2022 Aug 31;13:999886. doi: 10.3389/fmicb.2022.999886 (PMC9471092; doi:10.3389/fmicb.2022.999886)

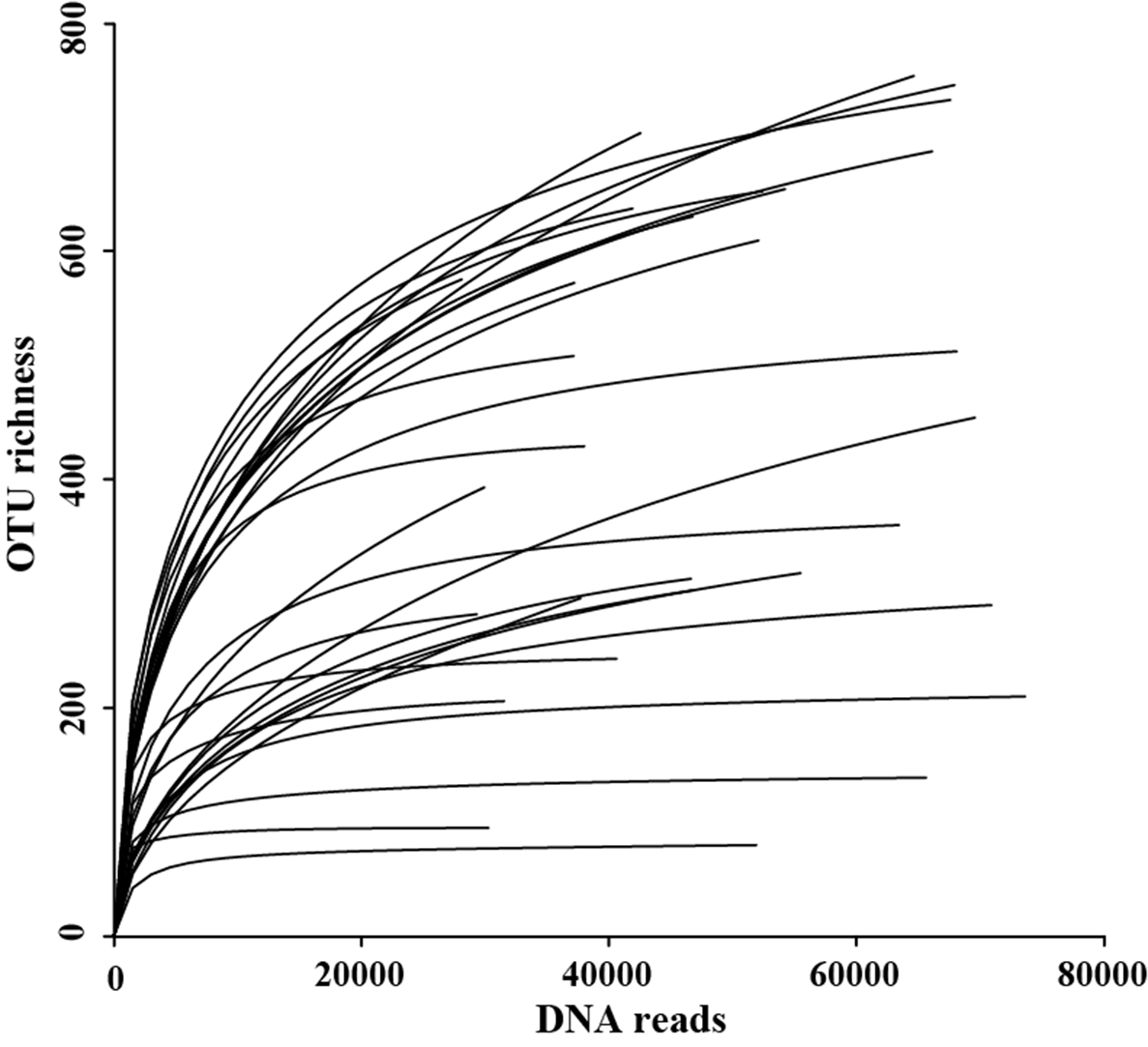

Supplement: Supplementary Figure 1 — Rarefaction curves of DNA reads based on OTUs at 97% sequence similarity. [file Image_1.TIF]
