# Supplementary material for: Metabarcoding of harmful algal bloom species in sediments from four coastal areas of the southeast China
Source: Front Microbiol. 2022 Aug 31;13:999886. doi: 10.3389/fmicb.2022.999886 (PMC9471092; doi:10.3389/fmicb.2022.999886)
Supplement: Supplementary file 2 [file Table_1.DOCX]

Supplementary Table 1 The taxonomic status, ecological information, distribution, and abundance of identified algal species

| Taxonomy | Species identified | Habitat type^1^ | HAB species | Resting stage | DF | XS | FN | DS | Level of abundance | OTUs |
| --- | --- | --- | --- | --- | --- | --- | --- | --- | --- | --- |
| Bacillariophyta |  |  |  |  |  |  |  |  |  |  |
| Bacillariophyceae | *Asterionellopsis glacialis* | Marine | Bloom (Roerig and Garcia., 2003) | * ^5^ |  |  | + |  | rare | OTU1673 |
|  | *Entomoneis ornata* | Marine |  |  |  |  | + |  | rare | OTU2560 |
|  | *Haslea nipkowii* | Freshwater |  |  | + |  |  |  | rare | OTU244 |
|  | *Navicula cryptocephala* | Marine/Freshwater |  |  | + |  |  |  | intermediate | OTU148 |
|  | *Navicula veneta* | Brackish |  |  |  | + |  | + | rare | OTU2379 |
|  | *Nitzschia palea* | Freshwater |  |  | + |  | + |  | intermediate | OTU298 |
|  | *Parlibellus delognei* f. *ellipticus* | Marine |  |  |  |  |  | + | abundant | OTU3608 |
|  | *Psammodictyon constrictum* | Marine |  |  |  |  |  | + | intermediate | OTU917 |
|  | *Pseudo-nitzschia micropora* | Marine |  |  |  | + | + | + | intermediate | OTU737 |
| Coscinodiscophyceae | *Guinardia delicatula* | Marine | Bloom^2^ |  |  | + |  | + | rare | OTU890 |
|  | *Paralia longispina* | Marine |  |  |  | + | + | + | intermediate | OTU3365 |
| Mediophyceae | *Arcocellulus cornucervis* | Marine |  |  | + |  | + | + | abundant | OTU3354, OTU3395 |
|  | *Cerataulina daemon* | Marine |  |  |  | + |  |  | rare | OTU1799 |
|  | *Chaetoceros anastomosans* | Marine |  | * (Oku and Kamatani., 1997) |  |  |  | + | intermediate | OTU3459 |
|  | *Chaetoceros* cf. *lauderi* | Marine |  | * ^4^ |  |  | + | + | intermediate | OTU1648 |
|  | *Chaetoceros costatus* | Marine |  | * ^4^ |  |  |  | + | intermediate | OTU2519 |
|  | *Chaetoceros curvisetus* | Marine | Bloom^2^ | * ^4^ | + |  | + | + | abundant | OTU3581, OTU3653 |
|  | *Chaetoceros debilis* | Marine | Bloom^2^ | * ^4^ | + | + | + | + | abundant | OTU3743 |
|  | *Chaetoceros didymus* | Marine |  | * ^4^ |  | + |  |  | intermediate | OTU1761 |
|  | *Chaetoceros elegans* | Marine |  | * (Li et al., 2017) |  |  |  | + | rare | OTU3571 |
|  | *Chaetoceros seiracanthus* | Marine |  | * ^4^ |  |  |  | + | rare | OTU952 |
|  | *Chaetoceros socialis* | Marine | Bloom^2^ | * ^5^ |  | + | + | + | abundant | OTU3701 |
|  | *Chaetoceros sporotruncatus* | Marine |  | * (Gaonkar et al., 2017) |  |  | + | + | abundant | OTU2580 |
|  | *Chaetoceros tenuissimus* | Marine |  | * ^5^ |  |  | + | + | intermediate | OTU3328 |
|  | *Conticribra guillardii* | Marine |  |  |  |  | + |  | rare | OTU901 |
|  | *Cyclotella striata* | Freshwater |  | * (Gao et al., 2017) |  | + |  | + | abundant | OTU2274 |
|  | *Ditylum sol* | Marine |  |  |  |  | + |  | intermediate | OTU1110 |
|  | *Leptocylindrus danicus* | Marine | Bloom (Karthik et al., 2017) | * ^5^ |  |  | + |  | intermediate | OTU1652 |
|  | *Minidiscus comicus* | Marine |  |  | + |  | + | + | abundant | OTU2585 |
|  | *Minidiscus proschkinae* | Marine |  |  |  | + | + | + | abundant | OTU1614 |
|  | *Skeletonema costatum* | Marine | Bloom^2^ | * ^5^ | + | + | + | + | abundant | OTU954 |
|  | *Skeletonema menzelii* | Marine |  | * ^5^ |  |  | + |  | intermediate | OTU1057 |
|  | *Skeletonema subsalsum* | Marine |  | * ^5^ | + | + | + | + | abundant | OTU1608 |
|  | *Thalassiosira aestivalis* | Marine | Bloom (Waite et al., 1992) |  |  |  | + | + | abundant | OTU926 |
|  | *Thalassiosira angulata* | Marine | Bloom (Radchenko et al., 2018) | * (Mills et al., 2006) | + | + | + | + | abundant | OTU3657 |
|  | *Thalassiosira tenera* | Marine |  |  | + |  | + | + | abundant | OTU1640 |
|  | *Trieres regia* | Marine | Bloom (Sun and Zhang., 2005) |  |  |  | + | + | rare | OTU2499 |
| Chlorophyta |  |  |  |  |  |  |  |  |  |  |
| Chlorodendrophyceae | *Tetraselmis marina* | Marine |  |  | + | + | + | + | abundant | OTU2781 |
|  | *Tetraselmis subcordiformis* | Marine |  |  | + | + |  |  | intermediate | OTU393 |
| Chlorophyceae | *Chlamydomonas kuwadae* | Marine/Freshwater |  |  |  | + |  |  | abundant | OTU1758 |
|  | *Chlamydomonas noctigama* | Freshwater/Terrestrial |  |  |  | + |  |  | intermediate | OTU2246 |
|  | *Chlorococcum oleofaciens* | Terrestrial |  |  | + |  |  | + | abundant | OTU91 |
|  | *Chloromonas reticulata* | Freshwater |  |  |  | + |  | + | abundant | OTU1745 |
|  | *Chlorosarcinopsis bastropiensis* | Terrestrial |  |  |  | + |  |  | intermediate | OTU2355 |
|  | *Floydiella terrestris* | Terrestrial |  |  |  | + |  |  | intermediate | OTU2284 |
|  | *Heterochlamydomonas callunae* | Terrestrial |  |  |  | + |  |  | abundant | OTU2038 |
|  | *Monoraphidium convolutum* | Freshwater |  |  | + | + |  | + | abundant | OTU3051 |
|  | *Mychonastes homosphaera* | Terrestrial |  |  |  | + |  | + | intermediate | OTU2163 |
|  | *Pediastrum duplex* | Freshwater |  |  |  | + | + | + | abundant | OTU3220 |
|  | *Pseudodidymocystis planctonica* | Freshwater |  |  | + |  |  | + | abundant | OTU3041 |
|  | *Rotundella rotunda* | Terrestrial |  |  |  | + |  |  | intermediate | OTU1840 |
|  | *Tetradesmus obliquus* | Freshwater |  |  | + | + |  | + | intermediate | OTU314 |
| Chloropicophyceae | *Chloroparvula japonica* | Marine |  |  | + |  |  | + | rare | OTU3318 |
| Mamiellophyceae | *Crustomastix stigmatica* | Marine |  |  | + | + | + | + | intermediate | OTU1896, OTU2230 |
|  | *Dolichomastix tenuilepis* | Marine |  |  |  | + | + |  | intermediate | OTU1302 |
|  | *Mamiella gilva* | Marine |  |  | + | + | + | + | abundant | OTU3129 |
|  | *Micromonas pusilla* | Marine | Bloom (Ismael., 2014) |  | + | + | + | + | abundant | OTU111 |
|  | *Ostreococcus lucimarinus* | Marine |  |  |  | + |  | + | intermediate | OTU3288 |
| Nephroselmidophyceae | *Nephroselmis pyriformis* | Marine | Bloom (Liu et al., 2020a) |  |  | + |  | + | intermediate | OTU1844 |
|  | *Nephroselmis rotunda* | Marine |  |  | + | + |  |  | abundant | OTU35 |
| Pedinophyceae | *Marsupiomonas pelliculata* | Brackish |  |  |  |  |  | + | intermediate | OTU3630 |
|  | *Pedinomonas minor* | Freshwater |  |  |  | + |  |  | intermediate | OTU1879 |
| Pyramimonadophyceae | *Prasinopapilla vacuolata* |  |  |  |  |  | + | + | intermediate | OTU3761 |
|  | *Pterosperma cristatum* | Marine |  |  | + | + | + | + | intermediate | OTU155 |
|  | *Pycnococcus provasolii* | Marine |  |  | + |  | + | + | intermediate | OTU3386 |
| Trebouxiophyceae | *Chloroidium saccharophilum* | Freshwater/Terrestrial |  |  | + |  |  | + | intermediate | OTU3578 |
|  | *Coccomyxa simplex* | Freshwater/Terrestrial |  |  | + | + |  | + | abundant | OTU3529 |
|  | *Myrmecia astigmatica* | Terrestrial |  |  |  |  |  | + | abundant | OTU3754 |
|  | *Oocystis marssonii* | Freshwater |  |  |  | + |  | + | abundant | OTU3680 |
|  | *Picochlorum atomus* | Marine |  |  |  | + | + | + | abundant | OTU2555 |
|  | *Picochlorum maculatum* | Marine |  |  | + | + | + | + | abundant | OTU2666 |
|  | *Symbiochloris irregularis* | Terrestrial |  |  | + |  |  |  | abundant | OTU591 |
|  | *Symbiochloris symbiontica* | Terrestrial |  |  |  |  |  | + | rare | OTU3617 |
|  | *Trebouxia aggregata* | Terrestrial |  |  | + |  |  |  | intermediate | OTU386 |
| Ulvophyceae | *Bryopsis plumosa* | Marine |  |  |  |  |  | + | intermediate | OTU3044 |
|  | *Planophila laetevirens* | Terrestrial |  |  |  | + |  |  | intermediate | OTU2317 |
| Cryptophyta |  |  |  |  |  |  |  |  |  |  |
| Cryptophyceae | *Teleaulax amphioxeia* | Marine |  |  | + |  | + |  | intermediate | OTU1087 |
|  | *Teleaulax gracilis* | Marine |  |  |  |  | + |  | intermediate | OTU743 |
| Dinophyta |  |  |  |  |  |  |  |  |  |  |
| Dinophyceae | *Adenoides eludens* | Marine |  |  | + |  | + |  | rare | OTU475 |
|  | *Alexandrium hiranoi* | Marine | goniodomine A^3^ | * ^2^ |  |  | + |  | abundant | OTU1553 |
|  | *Alexandrium ostenfeldii* | Marine | Bloom/PSP^3^ | * (Mackenzie et al., 1996) |  | + | + |  | abundant | OTU1103 |
|  | *Alexandrium satoanum* | Marine |  |  |  |  | + |  | abundant | OTU664 |
|  | *Alexandrium tamarense* | Marine | Bloom/PSP^3^ | * ^6^ |  | + | + |  | abundant | OTU1077 |
|  | *Amphidiniopsis arenaria* | Marine |  |  |  | + | + | + | abundant | OTU2466 |
|  | *Amylax triacantha* | Marine | Bloom (Rodríguez-Palacio et al., 2019) |  |  |  | + |  | intermediate | OTU996 |
|  | *Archaeperidinium minutum* | Marine |  | * (Mertens et al., 2012) |  | + | + |  | intermediate | OTU1797 |
|  | *Azadinium poporum* | Marine | Bloom/AZA^3^ | * (Luo et al., 2016) | + |  | + |  | abundant | OTU1007 |
|  | *Azadinium trinitatum* | marine |  | * (Luo et al., 2016) |  | + | + | + | abundant | OTU999 |
|  | *Barrufeta bravensis* | marine | Bloom (Sampedro et al., 2011) | * (Sampedro et al., 2011) | + | + | + | + | abundant | OTU791, OTU1343, OTU1419, OTU1516 |
|  | *Biecheleria tirezensis* | Marine |  | * (Raho et al., 2018) | + | + | + | + | abundant | OTU1288, OTU1538, OTU3398 |
|  | *Blixaea quinquecornis* | Marine | Bloom (Ismael., 2014) | * (Satta et al., 2010) |  |  |  | + | abundant | OTU3399 |
|  | *Dissodinium pseudolunula* | Marine |  | * (Gomez and Artigas., 2013) | + | + | + | + | abundant | OTU2889 |
|  | *Gonyaulax cochlea* | Marine |  |  | + | + | + |  | abundant | OTU1382, OTU1415 |
|  | *Gonyaulax ellegaardiae* | Marine |  | * (Mertens et al., 2015) |  |  | + | + | abundant | OTU3105 |
|  | *Gonyaulax spinifera* | Marine/Freshwater | Bloom/YTX ^2,3^ | * ^6^ | + | + | + | + | abundant | OTU1572, OTU1773, OTU2413 |
|  | *Gonyaulax whaseongensis* | Marine |  | * ^6^ |  |  |  | + | intermediate | OTU3497 |
|  | *Gotoius excentricus* | Marine |  |  |  |  | + | + | abundant | OTU3242 |
|  | *Gymnodinium corollarium* | Brackish | Bloom (Suikkanen et al., 2011) | * (Sundstrom et al., 2009) |  |  | + |  | intermediate | OTU710, OTU989 |
|  | *Gymnodinium impudicum* | Marine | Bloom (Park et al., 2010) | * (Park et al., 2010) |  | + | + | + | intermediate | OTU3532 |
|  | *Gymnodinium microreticulatum* | Marine |  | * ^2^ |  | + | + | + | abundant | OTU1705 |
|  | *Gymnodinium plasticum* | freshwater |  | * (Wang et al., 2017) |  | + | + | + | abundant | OTU666 |
|  | *Heterocapsa niei* | Marine | Bloom/Toxic (Reifel et al., 2002) |  | + | + | + | + | abundant | OTU2064 |
|  | *Islandinium tricingulatum* | Marine |  | * (Kawami et al., 2009) |  |  | + |  | intermediate | OTU1333 |
|  | *Karlodinium veneficum* | Marine | Bloom/Karlotoxins^3^ | * (Liu et al., 2020b) |  | + | + | + | abundant | OTU1597 |
|  | *Levanderina fissa* | Marine | Bloom^2^ | * ^6^ |  |  | + |  | abundant | OTU1349 |
|  | *Niea acanthocysta* | Marine |  | * (Liu et al., 2015) |  | + | + | + | abundant | OTU1129, OTU1217, OTU1719, OTU2673, OTU3719, OTU3830 |
|  | *Paragymnodinium asymmetricum* | Marine |  |  | + | + | + | + | abundant | OTU2073 |
|  | *Polykrikos hartmannii* | marine | Bloom/Ichthyotoxic ^3^ | * ^6^ |  | + | + | + | abundant | OTU605,OTU1360 |
|  | *Polykrikos kofoidii* | Marine |  | * ^6^ |  |  | + |  | intermediate | OTU983 |
|  | *Polykrikos schwartzii* | Brackish |  | * ^6^ |  |  | + | + | abundant | OTU2733 |
|  | *Prorocentrum triestinum* | Marine | Bloom (Ndhlovu et al., 2017) |  |  | + | + | + | abundant | OTU1876 |
|  | *Protoceratium reticulatum* | Marine | Bloom/YTX ^3^ | * ^6^ |  |  | + |  | intermediate | OTU1573 |
|  | *Protodinium simplex* | Marine/Freshwater | Bloom (Luo et al., 2015) |  |  |  | + |  | rare | OTU1209 |
|  | *Protoperidinium abei* | Marine |  | * ^6^ |  |  | + |  | abundant | OTU1568 |
|  | *Protoperidinium claudicans* | Marine |  | * ^6^ |  |  | + | + | abundant | OTU3140, OTU3197, OTU3409, OTU3415 |
|  | *Protoperidinium denticulatum* | Marine |  | * ^6^ | + |  | + | + | abundant | OTU906, OTU2929, OTU3165 |
|  | *Protoperidinium leonis* | Marine |  | * ^6^ |  |  | + | + | abundant | OTU891, OTU3276, OTU3279, OTU3280, OTU3305, OTU3352, OTU3406, OTU3448, OTU3480, OTU3483, OTU3501, OTU3509, OTU3511, OTU3778, OTU3808, OTU3811 |
|  | *Protoperidinium monovelum* | Marine |  | * ^6^ |  | + | + | + | abundant | OTU3400 |
|  | *Protoperidinium pentagonum* | Marine |  | * ^6^ |  |  | + |  | abundant | OTU631, OTU649, OTU686, OTU725, OTU742, OTU752, OTU812 |
|  | *Pseudocochlodinium profundisulcus* | marine | Bloom/Ichthyotoxic (Shen et al., 2012) | * (Hu et al., 2021) |  | + | + |  | abundant | OTU1513 |
|  | *Scrippsiella acuminata* | Marine | Bloom^2^ | * ^6^ | + | + | + | + | abundant | OTU756, OTU1613, OTU1636, OTU1704, OTU2775, OTU3154, OTU3403, OTU3810 |
|  | *Sourniaea diacantha* | Marine |  | * (Zhang et al., 2020) |  |  |  | + | intermediate | OTU2657,OTU2761 |
|  | *Syltodinium listii* | Marine |  |  |  |  | + | + | abundant | OTU1476 |
|  | *Tripos fusus* | Marine |  |  |  | + | + |  | abundant | OTU1999 |
| Ellobiophyceae | *Ellobiopsis chattonii* | Marine |  |  |  | + | + | + | intermediate | OTU2591 |
| Noctilucophyceae | *Noctiluca scintillans* | Marine | Bloom ^2^ |  | + | + | + | + | abundant | OTU3422 |
| Syndiniophyceae | *Euduboscquella costata* | Marine |  |  |  | + | + |  | intermediate | OTU1361 |
| Haptophyta |  |  |  |  |  |  |  |  |  |  |
| Coccolithophyceae | *Chrysochromulina spinifera* | Marine |  |  |  |  | + |  | intermediate | OTU867 |
|  | *Phaeocystis jahnii* | Marine | Bloom ^2^ |  | + |  |  |  | abundant | OTU157 |
|  | *Tergestiella adriatica* | Marine |  |  |  |  | + |  | rare | OTU1126 |
| Katablepharidophyta |  |  |  |  |  |  |  |  |  |  |
| Katablepharidophyceae | *Katablepharis japonica* | Marine |  |  |  |  | + |  | intermediate | OTU1483 |
| Ochrophyta |  |  |  |  |  |  |  |  |  |  |
| Chrysophyceae | *Lepidochromonas butcheri* | Freshwater |  |  | + | + |  | + | abundant | OTU579 |
|  | *Paraphysomonas imperforata* | Brackish |  | * (Takahashi et al., 1986) | + |  | + | + | abundant | OTU600 |
|  | *Paraphysomonas lucasi* | Marine |  | * (Coradeghini and Vigna, 2008） | + |  |  | + | abundant | OTU580 |
| Dictyochophyceae | *Pseudopedinella elastica* | Brackish |  |  |  | + | + |  | abundant | OTU1820 |
| Eustigmatophyceae | *Nannochloropsis limnetica* | Freshwater |  |  | + | + | + | + | abundant | OTU571 |
|  | *Nannochloropsis oculata* | Brackish |  |  | + | + | + | + | abundant | OTU275 |
|  | *Vacuoliviride crystalliferum* | Freshwater |  |  | + |  |  | + | intermediate | OTU25 |
| Pelagophyceae | *Aureococcus anophagefferens* | Marine | Bloom/Ichthyotoxic ^2, 3^ | * (Tang et al., 2019) | + |  | + |  | intermediate | OTU213 |
| Raphidophyceae | *Chattonella marina* | Marine | Bloom/Ichthyotoxic ^3^ | * (Onitsuka et al., 2020) |  |  |  | + | intermediate | OTU3750 |
|  | *Chattonella subsalsa* | Marine | Bloom/Ichthyotoxic ^3^ | * (Onitsuka et al., 2020) | + | + | + |  | abundant | OTU2007 |
|  | *Fibrocapsa japonica* | Marine | Bloom/Ichthyotoxic ^3^ | * (Cucchiari et al., 2010) |  |  | + |  | rare | OTU1212 |
|  | *Heterosigma akashiwo* | Marine | Bloom/Ichthyotoxic ^3^ | * ^2^ | + |  | + |  | intermediate | OTU138 |
| Synurophyceae | *Poterioochromonas stipitata* | Freshwater |  |  | + | + | + |  | abundant | OTU1946 |
| Xanthophyceae | *Botrydiopsis callosa* | Terrestrial |  |  |  | + |  | + | intermediate | OTU2135 |
|  | *Characiopsis saccata* | Freshwater |  |  |  | + |  |  | abundant | OTU2028 |
|  | *Tribonema intermixum* | Freshwater |  |  |  | + |  |  | rare | OTU2290 |
| Rhodophyta |  |  |  |  |  |  |  |  |  |  |
| Florideophyceae | *Gracilariopsis chorda* | Marine |  |  |  |  |  | + | intermediate | OTU3470 |
| Rhodellophyceae | *Rhodella violacea* | Marine |  |  |  | + |  | + | intermediate | OTU3797 |
| Harmful species | | | | | 15 | 20 | 33 | 21 |  |  |
| resting species | | | | | 17 | 25 | 48 | 38 |  |  |
| Overall species | | | | | 53 | 76 | 95 | 91 |  |  |

*: Taxa reported to form resting stages. +: Occurrence in the three sea areas. DF: the Dafeng Port, XS: the Xiangshan Bay, FN: the Funing Bay, DS: the Dongshan Bay. HAB: Harmful algal bloom species, PSP: Paralytic shellfish poisoning, AZP: Azaspiracid shellfish poisoning, YTX: Yessotoxin. References, 1: Guiry and Guiry, 2021; 2: Hallegraeff et al., 2004; 3: Lundholm et al., 2009 onwards; 4: Ishii et al., 2011; 5: Montresor et al., 2013; 6: Matsuoka and Fukuyo, 2000. The abundant species were supported by at least one OTU of the species contributing 0.1% to the eukaryotic reads in at least one sample, the intermediate and rare OTUs were supported by < 0.1% but ≥ 0.01% of reads, and by < 0.01% of reads, respectively (Logares et al., 2014; Mangot et al., 2013).

References:

Coradeghini, A., and Vigna, M.S. (2008). The aerial epiphytic stomatocyst flora (Chrysophyceae and Synurophyceae) of mosses from Primavera Station, Antarctica. *Nova Hedwigia* 86, 401-417. doi: 10.1127/0029-5035/2008/0086-0401

Cucchiari, E., Pistocchi, R., Pezzolesi, L., Penna, A., Battocchi, C., Cerino, F., et al. (2010). Resting cysts of *Fibrocapsa japonica* (Raphidophyceae) from coastal sediments of the northern Adriatic Sea (Mediterranean Sea). *Harmful Algae* 10, 81-87. doi: 10.1016/j.hal.2010.07.003

Gao, Y., Deng, C., Chen, C., Liang, J., and Sun, L. (2017). Changes of sedimentary diatoms and resting spores over a 60 years period in the East China Sea and their environmental indication. *Phycologia* 56, 57-58.

Gaonkar, C.C., Kooistra, W.H.C.F., Lange, C.B., Montresor, M., and Sarno, D. (2017). Two new species in the *Chaetoceros socialis* complex (Bacillariophyta): *C. sporotruncatus* and *C. dichatoensis*, and characterization of its relatives, *C. radicans* and *C. cinctus*. *J. Phycol*. 53, 889-907. doi: 10.1111/jpy.12554

Gomez, F., and Artigas, L.F. (2013). The formation of the twin resting cysts in the dinoflagellate *Dissodinium pseudolunula*, a parasite of copepod eggs. *J. Plankton Res*. 35, 1167-1171. doi: 10.1093/plankt/fbt066

Guiry, M.D., and Guiry, G.M. (2021). AlgaeBase. World-wide Electronic Publication. National University of Ireland, Galway. http://www.algaebase.org.

Hallegraeff, G.M., Anderson, D.M., Cembella, A.D., and Enevoldsen, H.O. (2004). Manual on harmful marine microalgae. UNESCO, Paris, France.

Ishii, K.I., Iwataki, M., Matsuoka, K., and Imai, I. (2011). Proposal of identification criteria for resting spores of *Chaetoceros* species (Bacillariophyceae) from a temperate coastal sea. *Phycologia* 50, 351-362. doi: 10.2216/10-36.1

Ismael, A.A. (2014). Coastal engineering and harmful algal blooms along Alexandria coast, Egypt. *Egypt. J. Aquat. Res*. 40, 125-131. doi: 10.1016/j.ejar.2014.07.005

Karthik, R., Padmavati, G., Elangovan, S.S., and Sachithanandam, V. (2017). Monitoring the diatom bloom of *Leptocylindrus danicus* (Cleve 1889, Bacillariophyceae) in the coastal waters of South Andaman Island. *Indian J. Geo-Mar. sci*. 46, 958-965. http://nopr.niscpr.res.in/handle/123456789/41662

Kawami, H., Van Wezel, R., Koeman, R.P.T., and Matsuoka, K. (2009). *Protoperidinium tricingulatum* sp. nov. (Dinophyceae), a new motile form of a round, brown, and spiny dinoflagellate cyst. *Phycol. Res.* 57, 259-267. doi: 10.1111/j.1440-1835.2009.00545.x

Hu, Z., Xu, N., Gu, H., Chai, Z., Takahashi, K., Li, Z., Deng, Y., Iwataki, M., Matsuoka, K., Tang, Y. (2021). Morpho-molecular description of a new HAB species, *Pseudocochlodinium profundisulcus* gen. et sp. nov., and its LSU rRNA gene based genetic diversity and geographical distribution. *Harmful Algae* 108, 102098. doi: 10.1016/j.hal.2021.102098

Li, Y., Boonprakob, A., Gaonkar, C.C., Kooistra, W.H.C.F., Lange, C.B., Hernández-Becerril, D., et al. (2017). Diversity in the globally distributed diatom genus *Chaetoceros* (Bacillariophyceae): three new species from warm-temperate waters. *PLoS One* 12: e0168887. doi: 10.1371/journal.pone.0168887

Liu, S., Gibson, K., Cui, Z., Chen, Y., Sun, X., and Chen, N. (2020a). Metabarcoding analysis of harmful algal species in Jiaozhou Bay. *Harmful algae* 92, 101772. doi: 10.1016/j.hal.2020.101772

Liu, T., Mertens, K.N., and Gu, H. (2015). Cyst-theca relationship and phylogenetic positions of the diplopsalioideans (Peridiniales, Dinophyceae), with description of *Niea* and *Qia gen. nov*.. *Phycologia* 54, 210-232. doi: 10.2216/14-94.1

Liu, Y., Hu, Z., Deng, Y., and Tang, Y. (2020b). Evidence for resting cyst production in the cosmopolitan toxic dinoflagellate Karlodinium veneficum and the cyst distribution in the China seas. *Harmful Algae*, 93, 101788. doi: 10.1016/j.hal.2020.101788

Logares, R., Audic, S., Bass, D., Bittner, L., Boutte, C., Christen, R., et al. (2014). Patterns of rare and abundant marine microbial eukaryotes. *Curr. Biol*. 24, 813-821. doi: 10.1016/j.cub.2014.02.050

Lundholm, N., Churro, C., Fraga, S., Hoppenrath, M., Iwataki, M., Larsen, J., Mertens, K., Moestrup, and Ø., Zingone, A. (Eds) (2009 onwards). IOC-UNESCO Taxonomic Reference List of Harmful Micro Algae. Accessed at https://www.marinespecies.org/hab on 2022-03-11. doi:10.14284/362

Luo, Z., Yang, W., Xu, B., Zheng, B., and Gu, H. (2015). Morphology, ultrastructure, and phylogeny of *Protodinium simplex* and *Biecheleriopsis cf. adriatica* (Dinophyceae) from the China Sea. *Nova Hedwigia* 101, 251-268. doi: 10.1127/nova_hedwigia/2015/0268

Luo, Z., Krock, B., Mertens, K.N., Price, A.M., Turner, R.E., Rabalais, N.N., et al. (2016). Morphology, molecular phylogeny and azaspiracid profile of *Azadinium poporum* (Dinophyceae) from the Gulf of Mexico. *Harmful Algae* 55, 56-65. doi: 10.1016/j.hal.2016.02.006

Mackenzie, L., White, D., Oshima, Y., and Kapa, J. (1996). The resting cyst and toxicity of *Alexandrium ostenfeldii* (Dinophyceae) in New Zealand. *Phycologia* 35, 148-155. doi: 10.2216/i0031-8884-35-2-148.1

Mangot, J. F., Domaizon, I., Taib, N., Marouni, N., Duffaud, E., Bronner, G., et al. (2013). Short-term dynamics of diversity patterns: evidence of continual reassembly within lacustrine small eukaryotes. *Environ. Microbiol*. 15, 1745-1758. doi: 10.1111/1462-2920.12065

Matsuoka, K., and Fukuyo, Y. (2000). Technical guide for modern dinoflagellate cyst study. WESTPAC-HAB, Japan Society for the Promotion of Science, Tokyo, Japan.

Mertens, K.N., Yamaguchi, A., Kawami H., Ribeiro, S., Leander, B.S., Price, A.M., et al. (2012). *Archaeperidinium saanichi* sp. nov.: A new species based on morphological variation of cyst and theca within the *Archaeperidinium minutum* Jörgensen 1912 species complex. *Mar. Micropaleontol*. 96, 48-62. doi: 10.1016/j.marmicro.2012.08.002

Mertens, K.N., Aydin, H., Uzar, S., Takano, Y., Yamaguchi, A., and Matsuoka, K. (2015). Relationship between the dinoflagellate cyst *Spiniferites pachydermus* and *Gonyaulax ellegaardiae* sp. nov. from Izmir Bay, Turkey, and molecular characterization. *J. Phycol*. 51, 560-573. doi: 10.1111/jpy.12304

Mills, K.E., and Kaczmarska, I. (2006). Autogamic reproductive behavior and sex cell structure in *Thalassiosira angulata* (Bacillariophyta). *Bot. Mar.* 49, 417-430. doi: 10.1515/BOT.2006.053

Montresor, M., Di Prisco, C., Sarno, D., Margiotta, F., and Zingone, A. (2013). Diversity and germination patterns of diatom resting stages at a coastal Mediterranean site. *Mar. Ecol.: Prog. Ser.* 484, 79-95. doi: 10.3354/meps10236

Ndhlovu, A., Dhar, N., Garg, N., Xuma, T., Pitcher, G.C., Sym, S.D., et al. (2017). A red tide forming dinoflagellate *Prorocentrum triestinum*: identification, phylogeny and impacts on St Helena Bay, South Africa. *Phycologia* 56, 649-665. doi: 10.2216/16-114.1

Oku, O., and Kamatani, A. (1997). Resting spore formation of the marine planktonic diatom *Chaetoceros anastomosans* induced by high salinity and nitrogen depletion. *Mar. biol.* 127, 515-520. doi: 10.1007/s002270050040

Onitsuka, G., Yamaguchi, M., Sakamoto, S., Shikata, T., Nakayama, N., Kitatsuji, S., et al. (2020). Interannual variations in abundance and distribution of *Chattonella* cysts, and the relationship to population dynamics of vegetative cells in the Yatsushiro Sea, Japan. *Harmful Algae* 9, 101833. doi: 10.1016/j.hal.2020.101833

Park, T.G., and Park, Y.T. (2010). Detection of *Cochlodinium polykrikoides* and *Gymnodinium impudicum* (Dinophyceae) in sediment samples from Korea using real-time PCR. *Harmful Algae* 9, 59-65. doi: 10.1016/j.hal.2009.08.002

Radchenko, I.G., Shevchenko, V.P., Kravchishina, M.D., Il’inskii, V.V., Georgiev, A.P., Tolstikov, A.V., et al. (2018). The First Record of *Thalassiosira angulata* (Bacillariophyceae) Bloom in the White Sea: Spatial Distribution and Associated Species. *Moscow Univ. Biol. Sci. Bull*. 73, 217-221. doi: 10.3103/S0096392518040089

Raho, N., Fraga, S., Abad, J.P., and Marín, I. (2018). *Biecheleria tirezensis* sp. nov. (Dinophyceae, Suessiales), a new halotolerant dinoflagellate species isolated from the athalassohaline Tirez natural pond in Spain. *Eur. J. Phycol.* 53, 99-115. doi: 10.1080/09670262.2017.1386328

Reifel, K.M., McCoy, M.P., Rocke, T.E., Tiffany, M.A., Hurlbert, S.H., and Faulkner, D.J. (2002). Possible importance of algal toxins in the Salton Sea, California. *Hydrobiologia* 473, 275-292. doi: 10.1023/A:1016518825934

Rodríguez-Palacio, M., Lozano-Ramírez, C., and Alvarez-Hernández, S.H. (2019). HABs (Harmful Algal Blooms) Analysis, Their Cost, and Ecological Consequences. In: Ibáñez, A. (eds) Mexican Aquatic Environments. Springer, Cham. doi: 10.1007/978-3-030-11126-7_10

Roerig, L.R., and Garcia, V.M.T. (2003). Accumulations of the surf-zone diatom *Asterionellopsis glacialis* (Castracane) Round in Cassino Beach, Southern Brazil, and its relationship with environmental factors. *J. Coastal Res*. SI 35, 167-177. https://www.jstor.org/stable/40928758

Sampedro, N., Fraga, S., Penna, A., Casabianca, S., Zapata, M., Grünewald, C.F., et al. (2011). *Barrufeta bravensis* gen. Nov. Sp. Nov. (dinophyceae): A new bloom-forming species from the northwest mediterranean sea. *J. Phycol.* 47, 375-393. doi: 10.1111/j.1529-8817.2011.00968.x

Satta, C.T., Anglès S., Garcés, E., Lugliè, A., Padedda, B.M., and Sechi, N. (2010). Dinoflagellate cysts in recent sediments from two semi-enclosed areas of the Western Mediterranean Sea subject to high human impact. *Deep Sea Res., Part II* 57, 256-267. doi: 10.1016/j.dsr2.2009.09.013

Shen, P., Li, Y., Qi, Y., Zhang, L., Tan, Y., and Huang, L. (2012). Morphology and bloom dynamics of *Cochlodinium geminatum* (Schütt) Schütt in the Pearl River Estuary, South China Sea. *Harmful algae* 13, 10-19. doi: 10.1016/j.hal.2011.09.009

Suikkanen, S., Hakanen, P., Spilling, K., and Kremp, A. (2011). Allelopathic effects of Baltic Sea spring bloom dinoflagellates on co-occurring phytoplankton. *Mar. Ecol.: Prog.* *Ser*. 439, 45-55. doi: 10.3354/meps09356

Wu, Y., Sun, S., and Zhang, Y. (2005). Long-time change of environment and it's influence on phytoplankton community structure in Jiaozhou bay. *Oceanol. Limnol. Sin*. 36, 487-498.

Sundström, A.M., Kremp, A., Daugbjerg, N., Moestrup, Ø., Ellegaard, M., Hansen, R., et al., (2009). *Gymnodinium corollarium* sp. nov.(dinophyceae)—a new cold‐water dinoflagellate responsible for cyst sedimentation events in the baltic sea. *J. Phycol.* 45, 938-952. doi: 10.1111/j.1529-8817.2009.00712.x

Takahashi, E., Watanabe, K., and Satoh, H. (1986). Siliceous cysts from Kita-no-seto Strait, north of Syowa Station, Antarctica. *Mem. Natl. Inst. Polar Res.* 40, 84-95.

Tang, Y., Ma, Z., Hu, Z., Deng, Y., Yang, A., Lin, S., et al. (2019). 3,000 km and 1,500-year presence of *Aureococcus anophagefferens* reveals indigenous origin of brown tides in China. *Mol. Ecol.* 28, 4065-4076. doi: 10.1111/mec.15196

Waite, A., Bienfang, P.K., and Harrison, P.J. (1992). Spring bloom sedimentation in a subarctic ecosystem. *Mar. Biol.* 114, 119-129. doi: 10.1007/BF00350861

Wang, N., Luo, Z., Mertens, K.N., McCarthy, F.M.G., Gu, L., and Gu, H. (2017). Cyst‐motile stage relationship and molecular phylogeny of a new freshwater dinoflagellate *Gymnodinium plasticum* from Plastic Lake, Canada. *Phycol. Res*. 65, 312-321. doi: 10.1111/pre.12190

Zhang, W., Li, Z., Mertens, K.N., Derrien, A., Pospelova, V., Carbonell-Moore, M.C., et al. (2020). Reclassification of *Gonyaulax verior* (Gonyaulacales, Dinophyceae) as *Sourniaea diacantha gen. et comb. nov*.. *Phycologia* 59, 246-261. doi: 10.1080/00318884.2020.1735926
