# Supplementary material for: Metabarcoding of harmful algal bloom species in sediments from four coastal areas of the southeast China
Source: Front Microbiol. 2022 Aug 31;13:999886. doi: 10.3389/fmicb.2022.999886 (PMC9471092; doi:10.3389/fmicb.2022.999886)
Supplement: Supplementary file 3 [file Table_2.DOCX]

**Supplementary Table 2** Biogenic elements in surface sediments from the four sea areas

| Station | TOC (%) | TN (%) | TP (%) | BSi (%)） | MC (%) |
| --- | --- | --- | --- | --- | --- |
| DF1 | 0.69 | 0.12 | 0.073 | 0.99 | 24.34 |
| DF2 | 0.60 | 0.09 | 0.066 | 0.78 | 20.39 |
| DF3 | 0.86 | 0.12 | 0.070 | 1.08 | 24.13 |
| DF4 | 1.06 | 0.13 | 0.070 | 1.14 | 23.88 |
| DF5 | 0.78 | 0.13 | 0.064 | 0.93 | 25.59 |
| DF6 | 0.92 | 0.11 | 0.054 | 0.90 | 25.63 |
| DF7 | 0.87 | 0.12 | 0.061 | 0.84 | 25.08 |
| **Ave DF** | **0.83** | **0.12** | **0.065** | **0.95** | **24.15** |
| XS1 | 1.29 | 0.14 | 0.034 | 1.32 | 31.97 |
| XS2 | 1.38 | 0.20 | 0.065 | 1.43 | 37.06 |
| XS3 | 1.39 | 0.16 | 0.037 | 1.28 | 36.23 |
| XS4 | 1.30 | 0.14 | 0.038 | 1.33 | 34.24 |
| XS5 | 1.26 | 0.16 | 0.044 | 1.70 | 37.10 |
| XS6 | 1.46 | 0.26 | 0.038 | 1.59 | 34.85 |
| XS7 | 1.27 | 0.21 | 0.036 | 1.43 | 31.05 |
| **Ave XS** | **1.34** | **0.18** | **0.042** | **1.44** | **34.64** |
| FN1 | 1.13 | 0.28 | 0.020 | 0.70 | 32.65 |
| FN2 | 1.13 | 0.26 | 0.021 | 1.36 | 33.33 |
| FN3 | 1.22 | 0.26 | 0.021 | 1.29 | 52.55 |
| FN4 | 1.44 | 0.33 | 0.029 | 1.69 | 43.80 |
| FN5 | 1.52 | 0.30 | 0.027 | 1.67 | 42.70 |
| FN6 | 1.37 | 0.33 | 0.032 | 1.63 | 42.70 |
| FN7 | 1.42 | 0.31 | 0.032 | 1.52 | 42.47 |
| **Ave FN** | **1.32** | **0.29** | **0.026** | **1.41** | **41.46** |
| DS1 | 1.28 | 0.19 | 0.063 | 1.48 | 48.31 |
| DS2 | 1.39 | 0.21 | 0.074 | 1.02 | 41.92 |
| DS3 | 1.24 | 0.20 | 0.063 | 1.18 | 38.75 |
| DS4 | 1.22 | 0.19 | 0.063 | 1.34 | 43.95 |
| DS5 | 1.21 | 0.19 | 0.063 | 1.30 | 40.17 |
| DS6 | 1.27 | 0.19 | 0.063 | 1.08 | 50.87 |
| DS7 | 1.23 | 0.21 | 0.069 | 1.40 | 45.83 |
| DS8 | 1.12 | 0.16 | 0.054 | 1.24 | 52.89 |
| DS9 | 1.32 | 0.21 | 0.070 | 0.80 | 42.24 |
| **Ave DS** | **1.25** | **0.20** | **0.065** | **1.20** | **44.99** |

Stations DF1-DF7 are in Dafeng Port (DF), stations XS1-XS7 in Xingshan Bay (XS), stations FN1-FN7 in Funing Bay (FN), and stations DS1-DS9 in Dongshan Bay (DS). TOC: total organic carbon, TN: total nitrogen, TP: total phosphorus, MC: moisture content.
